# *In vitro* hair growth-promoting effects of araliadiol via the p38/PPAR-γ signaling pathway in human hair follicle stem cells and dermal papilla cells

**DOI:** 10.3389/fphar.2024.1482898

**Published:** 2024-12-03

**Authors:** Seokmuk Park, Han Woong Park, Dae Bang Seo, Dae Sung Yoo, Seunghee Bae

**Affiliations:** ^1^ Department of Cosmetics Engineering, Konkuk University, Seoul, Republic of Korea; ^2^ ASK Company Co., Ltd., Daegu, Republic of Korea

**Keywords:** alopecia, araliadiol, hair follicles, hair growth, hair loss, phytochemicals, polyacetylene, PPAR-γ

## Abstract

**Background:**

Scalp hair plays a crucial role in social communication by expressing personal appearance and self-identity. Consequently, hair loss often leads to a perception of unattractiveness, negatively impacting an individual’s life and mental health. Currently, the use of Food and Drug Administration (FDA)-approved drugs for hair loss is associated with several side effects, highlighting the need for identifying new drug candidates, such as plant-derived phytochemicals, to overcome these issues.

**Objective:**

This study investigated the hair growth-promoting effects of araliadiol, a polyacetylene compound found in plants such as *Centella asiatica*.

**Methods:**

We employed an *in vitro* model comprising human hair follicle stem cells (HHFSCs) and human dermal papilla cells (HDPCs) to evaluate the hair growth-promoting effects of araliadiol. The proliferation-stimulating effects of araliadiol were assessed using water-soluble tetrazolium salt assay, adenosine triphosphate content assay, and crystal violet staining assay. In addition, we performed luciferase reporter assay, polymerase chain reaction analysis, cell fractionation, Western blot analysis, and enzyme-linked immunosorbent assay (ELISA) to elucidate the mechanism underlying the hair growth-inductive effects of araliadiol.

**Results:**

Araliadiol exhibited both proliferation- and hair growth-promoting effects in HHFSCs and HDPCs. Specifically, it increased the protein expression of cyclin B1 and Ki67. In HHFSCs, it elevated the expression of hair growth-promoting factors, including CD34, vascular endothelial growth factor (VEGF), and angiopoietin-like 4. Similarly, araliadiol increased the expression of hair growth-inductive proteins such as fibroblast growth factor 7, VEGF, noggin, and insulin-like growth factor 1 in HDPCs. Subsequent Western blot analysis and ELISA using inhibitors such as GW9662 and SB202190 confirmed that these hair growth-promoting effects were dependent on the p38/PPAR-γ signaling in both HHFSCs and HDPCs.

**Conclusion:**

Araliadiol promotes hair growth through the p38/PPAR-γ signaling pathway in human hair follicle cells. Therefore, araliadiol can be considered a novel drug candidate for the treatment of alopecia.

## Graphical Abstract

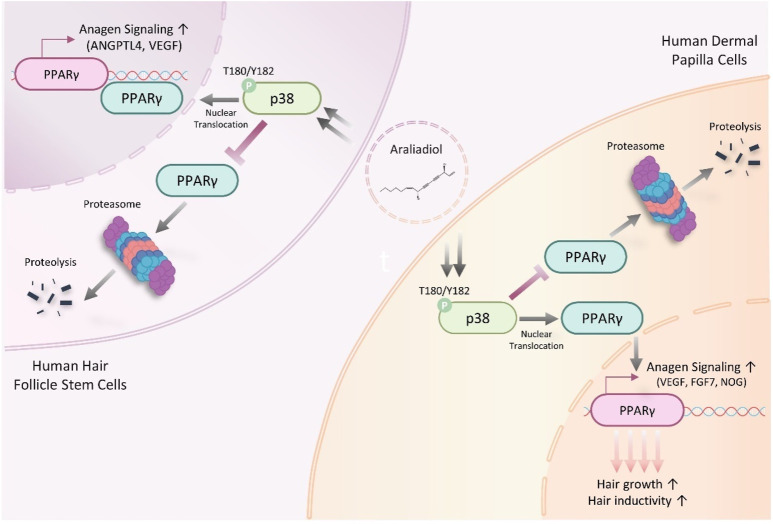

## 1 Introduction

Scalp hair is a unique human tissue, with its social and psychological importance often outweighing its biological significance. Hair serves as a prominent means of expressing body image and self-identity, significantly influencing perceptions of physical attractiveness (Patzer, 1988). Consequently, abundant hair is recognized as a sign of youth and vitality, whereas thinning hair and hair loss can lead to social disadvantages due to perceptions of unattractiveness (Dhami, 2021). Hence, individuals with hair loss often experience significant social and psychological stress, negatively impacting their quality of life (Grimalt, 2005). Previous studies have quantified the psychological impact of hair loss, reporting decreased self-esteem and an increased prevalence of psychological disorders in both men and women with alopecia (Williamson et al., 2001). In addition, about 40% of women with hair loss reported marital issues, and approximately 63% faced job-related problems, indicating that alopecia causes not only individual psychological stress but also broader social problems (Hunt and McHale, 2005).

Currently, the Food and Drug Administration (FDA)-approved drugs for alopecia include minoxidil, finasteride, cyproterone acetate, spironolactone, cyclosporine, corticosteroids, diphenylcyclopropenone, baricitinib, and ritlecitinib (Salim and Kamalasanan, 2020; Gupta et al., 2023; Gupta et al., 2024; Yan et al., 2024). In August 1988, the FDA approved minoxidil, branded as Rogaine, as the first treatment for androgenetic alopecia (Price, 1999). In 1997, finasteride was approved as the second treatment for androgenetic alopecia (Libecco and Bergfeld, 2004). Baricitinib and ritlecitinib were recently approved by the FDA in 2022 and 2023, respectively, for alopecia areata; both drugs work by inhibiting the action of Janus kinase (Gupta et al., 2023; Kincaid et al., 2023).

Although these synthetic drugs are globally used, numerous side effects and instances of ineffectiveness continue to be reported (Mounessa et al., 2023). Specifically, anti-androgenic drugs such as finasteride can cause side effects such as sexual dysfunction, including decreased libido, erectile dysfunction, and issues with orgasm (Irwig and Kolukula, 2011). Immunosuppressants, including baricitinib and ritlecitinib, commonly cause side effects such as infections, which can lead to conditions like herpes simplex, herpes zoster, and oral candidiasis (Ramírez-Marín and Tosti, 2023). In addition, minoxidil, the top-selling hair loss treatment, is known to cause mild symptoms such as erythema, scaling, pruritus, dermatitis, and itching, as well as systemic conditions, including cardiovascular side effects (Campese, 1981; Georgala et al., 2006; Rossi et al., 2012; Abdullah, 2020). Several recent studies have indicated the limited efficacy of minoxidil, with approximately 63.8% of users reporting no improvement or worsening of conditions and 59.3% experiencing at least one side effect (Shadi, 2023). Moreover, minoxidil requires at least 12 months of use, leading to a significantly low compliance rate. Given the side effects and limitations associated with currently used synthetic anti-hair loss drugs, there is a growing demand for the development of botanical drugs or cosmeceutical ingredients, such as plant-derived phytochemicals, as potential alternatives (Mccoy and Ziering, 2012; Asnaashari and Javadzadeh, 2020).

Phytochemicals (derived from the Greek word *phyto*, meaning plant) are constitutive metabolites that help plants withstand environmental challenges while supporting growth and reproduction (Molyneux et al., 2007). Recent research has emphasized the significant health benefits these compounds offer humans, functioning as substrates in biochemical reactions, enzyme cofactors, and ligands (Dillard and German, 2000; Chang et al., 2019). One of the primary advantages of using phytochemicals as drugs is their longstanding history as some of the oldest and most extensively utilized medicinal substances. Notably, over 50% of modern clinical drugs are derived from phytochemicals or rely on them as lead compounds, highlighting their crucial role in pharmaceutical development (Egbuna et al., 2019). Furthermore, phytochemicals have been rigorously studied for their bioactive properties, including antioxidant, antimicrobial, anti-inflammatory, anticancer, antiallergic, neuroprotective, and anti-aging effects (Saxena et al., 2013). Although synthetic drugs have advanced considerably in recent years and exhibit high efficacy, they often come with risks related to toxicity during metabolic processes. This has shifted attention toward phytomedicine, as certain phytochemicals have shown comparable effectiveness to synthetic drugs but with fewer or no side effects (Karimi et al., 2015; Meier and Lappas, 2016; Lappas et al., 2023). Additionally, the complex and costly nature of drug development—which currently requires investments over $1 billion—further underscores the potential of phytochemicals as promising drug candidates (Lupiáñez and Rufino-Palomares, 2021; Ahmed et al., 2024).

Polyacetylenes, or polyynes, are a type of phytochemical that contain two or more carbon-carbon triple bonds (R^1^−[CΞC]_n_−*R* ^2^) (Christensen, 1998). Over 2,500 natural polyacetylenes have been identified, and they are commonly found as secondary metabolites in organisms such as plants and algae (Wang et al., 2018; Christensen, 2020). These compounds are known to perform biological defense functions, such as antifungal activity, and have been reported to act as phytoalexins in plants (Christensen and Brandt, 2006; Mullins et al., 2021). Polyacetylene structures are biosynthesized from saturated fatty acids and, similar to other unsaturated organic substances, possess chemical and biological activities due to their conjugated triple bonds (Santos et al., 2022). Consequently, recent studies have elucidated different pharmacological effects of polyacetylene metabolites, including antifungal, anticancer, anti-inflammatory, and neuroprotective activities, with notable examples such as falcarinol, panaxydol, panaxytriol, and falcarindiol (Negri, 2015).

Araliadiol is a natural polyyne, an organic compound characterized by single and triple bonds. Initially discovered in *Aralia cordata*, recent studies have also identified its presence in *Centella asiatica* (*C. asiatica*) (Cheng et al., 2011; Fujimori et al., 2022). Polyacetylenes in *C. asiatica*, including araliadiol, have demonstrated therapeutic potential for several inflammatory diseases (Govindan et al., 2007; Fujimori et al., 2022; Jo et al., 2023). Specifically, araliadiol has been studied for its ability to enhance glucose uptake and protect against neuronal cell damage (Fujimori et al., 2022; Jo et al., 2023). *C. asiatica* has been traditionally used in India and other regions as a medicinal plant, with ethnomedicinal applications including hair growth promotion and anti-hair loss treatment (Biswas et al., 2021). Therefore, verifying the pharmacological efficacy of araliadiol, one of the polyacetylenic compounds present in *C. asiatica*, for promoting hair growth is significant.

This study, for the first time, investigated the potential of araliadiol as a drug candidate for the treatment of alopecia, as well as its underlying regulatory mechanisms.

## 2 Materials and methods

### 2.1 Chemicals

Araliadiol, purified from *C. asiatica* extract, was provided by ASK Company Co., Ltd. (Korea, Daegu). SB202190 (#HY-10295), a selective inhibitor of p38 MAP kinase, and GW9662 (#HY-16578), a PPAR-γ antagonist, were purchased from MedChemExpress (NJ, United States). MG132 (#474701) and cycloheximide (CHX; #01810) were obtained from Sigma-Aldrich (Merck KGaA; Darmstadt, Germany).

### 2.2 Preparation of *Centella asiatica* extract and isolation of araliadiol

*Centella asiatica* was harvested from Jeju Island, Korea. The plant material was washed five times with distilled water and air-dried at room temperature. Two kilograms of dried *C*. *asiatica* was extracted with 40 L of 70% methanol at 70°C for 5 h followed by centrifugation to collect the supernatant. The remaining residue was subjected to a second extraction under the same conditions with 40 L of 70% methanol for an additional 5 h. The supernatants from both extractions were combined, concentrated under reduced pressure using a Rotavapor R-100 rotary evaporator (Buchi, Flawil, Switzerland), and filtered through Whatman filter paper No. 1 (Whatman, Maidstone, UK) to obtain the final extract. The filtrate was concentrated under reduced pressure to eliminate methanol and then the resultant solution was partitioned between hexane and water. The hexane-soluble portion was concentrated *in vacuo* and subjected to silica gel column chromatography eluted with hexane-ethyl acetate (20:1→1:1, v/v, stepwise). An active fraction, H-3, was concentrated and separated by preparative reversed-phase MPLC eluted with a gradient of increasing amount of methanol in water, followed by Sephadex LH-20 column chromatography eluted with chloroform-methanol (1:1, v/v). Finally, an active fraction was subjected to preparative reversed-phase MPLC eluted with a gradient (50% aqueous methanol to methanol) to afford an active compound (28 mg) (Supplementary Figure S1).

### 2.3 High performance liquid chromatography analysis

Hitachi (Tokyo, Japan) High-Performance Liquid Chromatography Chromaster system equipped with a tunable UV-vis detector was used as the high performance liquid chromatography (HPLC) instrument. The chromatographic analysis was performed using a PAK C18 column (250 mm × 4.6 mm; OSAKA SODA, Japan). The elution conditions were as follows: column temperature, 25°C; flow rate, 1 mL/min; injection volume, 10 μL. The mobile phase consisted of solvent A (methanol) and solvent B (0.04% trifluoroacetic acid in water). Finally, detection was carried out using a UV detector set at a wavelength of 208 nm.

### 2.4 Cell culture for human hair follicle cells

To investigate the *in vitro* hair growth-promoting effect of araliadiol, the compound was applied to human hair follicle stem cells (HHFSCs; #36007–08; Celprogen, CA, United States) and human hair follicle dermal papilla cells (HDPCs; #C-12071; PromoCell, Heidelberg, Germany). Following the methods described in previous studies, HHFSCs and HDPCs were used at passages three to five and four to 7, respectively (Gupta et al., 2018; Wen et al., 2021; Park et al., 2024). The HHFSCs and HDPCs were cultured in T75 cm^2^ T-flasks (8 × 10^3^ cells/cm^2^) containing hair follicle stem cell undifferentiation media (#M36007-08US; Celprogen) and follicle growth medium (#C-26051; PromoCell), respectively. In addition, 293T cells (#CRL-3216) were obtained from the American Type Culture Collection (VA, United States) and cultured in Dulbecco’s modified Eagle’s medium (#LB001-05; Welgene, Seoul, Republic of Korea) supplemented with 10% (v/v) fetal bovine serum (#35–015-CV; Gibco; Thermo Fisher Scientific, MA, United States) and 1% penicillin/streptomycin (#15140–122; Gibco; Thermo Fisher Scientific). All cells were sub-cultured at 80% confluence and maintained at 37°C and 5% CO₂ in a humidified atmosphere.

### 2.5 Tetrazolium reduction assay

The cytotoxicity of araliadiol in 293T cells, HHFSCs, and HDPCs was evaluated using the water-soluble tetrazolium salt (WST-1) assay, with the EZ-Cytox Cell Viability Assay Kit (#EZ3000; DoGenBio, Seoul, Republic of Korea). Briefly, 293T cells (3 × 10^3^), HHFSCs (5 × 10^2^), and HDPCs cells (1 × 10^3^) were seeded in 96-well plates and incubated at 37°C for 24 h. Next, the cells were exposed to different concentrations of araliadiol (0–10 μg/mL) for up to 72 h. After treatment, the supernatant was discarded and 100 µL of the EZ-Cytox solution was added to each well. The plates were incubated at 37°C for 30 min, and cell viability was assessed by measuring the absorbance at 450 nm using a Synergy™ HTX Multi-Mode Microplate Reader (Bioteck; VT, United States).

### 2.6 Intracellular ATP detection assay

To assess the proliferative effects of araliadiol, an intracellular ATP detection assay was employed. HHFSCs (5 × 10^2^) and HDPCs (1 × 10^3^) were seeded in 96-well plates and incubated at 37°C for 24 h. Afterward, the cells were treated with the indicated concentrations of araliadiol (0–5 μg/mL) for 72 h. After treatment, the intracellular ATP content was measured using the CellTiter-Glo^®^ 2.0 Cell Viability Assay kit (#G9242; Promega, WI, United States). The reagent was directly added to each well, and the plates were mixed for 2 min on an orbital shaker to induce cell lysis. Subsequently, they were incubated in the dark at 25°C for 20 min to stabilize the luminescent signal. The luminescence signal was detected using the microplate reader.

### 2.7 Crystal violet staining assay

A crystal violet staining assay was performed to verify the proliferative effects of araliadiol on hair follicle cells. HHFSCs (3 × 10^4^) and HDPCs (6 × 10^4^) were seeded in 24-well plates and incubated at 37°C for 24 h. Different concentrations of araliadiol (0–2.5 μg/mL) were added to the plates, and the cells were incubated for an additional 72 h. After incubation, the cells were washed twice with 500 μL of Dulbecco’s phosphate buffered saline (DPBS). The residual DPBS was aspirated to ensure complete drying, and each well was stained with 0.5% crystal violet (#6408; BIOPURE, Gyeonggi-do, Republic of Korea) solution in 20% methanol for 30 min. The plates were rinsed five times with distilled water and allowed to air dry. The crystal violet solution retained within the cells was completely solubilized using 100% methanol. Proliferation rates were analyzed by measuring the absorbance at 570 nm using the microplate reader.

### 2.8 Transient transfection and luciferase reporter assay

To investigate the hair growth-enhancing effects of araliadiol, transient transfection and luciferase reporter assays were conducted using previously described methods (Park et al., 2024). The pSV-β-galactosidase plasmid (#E1081) was sourced from Promega, whereas the Gli response element-driven luciferase reporter plasmid (#113712), TCF/LEF response element-driven luciferase reporter plasmid (#12456), and VEGF promoter-driven luciferase reporter plasmid (#66128) were acquired from Addgene (MA, United States). For transient transfection, 293T cells (2 × 10⁵) were seeded in 6-well plates and incubated at 37°C for 24 h. Subsequently, each reporter plasmid was transfected using the Lipofectamine transfection reagent (#18324,012; Invitrogen; Thermo Fisher Scientific). The transfected cells were treated with the indicated concentrations of araliadiol (0–5 μg/mL) at 37°C for 48 h. Following treatment, the cells were lysed with passive lysis 5× buffer (#E1941; Promega) for 30 min at 4°C. Cell lysates were collected following centrifugation at 15,000 × g for 30 min at 4°C. D-luciferin (#88294; Invitrogen; Thermo Fisher Scientific) was added to the cell lysate and incubated at 37°C for 30 min, after which luciferase activity was determined using the microplate reader. β-Galactosidase activity was measured using the Beta-Glo Assay System (#E4720; Promega), and relative luciferase activity was normalized to β-galactosidase activity.

### 2.9 RNA extraction and polymerase chain reaction analysis

HHFSCs (2 × 10^5^) and HDPCs (3 × 10^5^) were seeded in 100 mm dishes and incubated at 37°C for 24 h. The cells were treated with araliadiol (0–2.5 μg/mL) for 48 h. Total RNA extraction and polymerase chain reaction (PCR) analysis were performed as described previously (Park et al., 2023a; Park et al., 2023b). Glyceraldehyde-3-phosphate dehydrogenase (*GAPDH*) was used as the reference for gene expression normalization. The following primers were used for PCR: *PPAR-γ*, 5′-CTC​ATG​AAG​AGC​CTT​CCA​ACT​CC-3’ (forward) and 5′-ACC​CTT​GCA​TCC​TTC​ACA​AGC-3’ (reverse); *GAPDH*, 5′-TCC​AAA​ATC​AAG​TGG​GGC​GAT​GC-3’ (forward) and 5′-GCC​AGT​AGA​GGC​AGG​GAT​GAT​GT-3’ (reverse).

### 2.10 Cell fractionation and Western blot analysis

HHFSCs (2 × 10^5^) and HDPCs (3 × 10^5^) were seeded in 100 mm dishes and incubated at 37°C for 24 h. The cells were treated with araliadiol (0–2.5 μg/mL) for 48 h, either in the presence or absence of CHX (two to five µM), MG132 (0.3–2.5 µM), SB202190 (20 µM), or GW9662 (20 µM). Total cell lysate was obtained using the RIPA protein lysis buffer, and nuclear and cytoplasmic fractions were separated using NE-PER reagents (Invitrogen; Thermo Fisher Scientific, MA, United States). The lysis buffer was supplemented with a protease inhibitor cocktail (#04693116001; Roche; Merck KGaA) and a phosphatase inhibitor cocktail (#4906845001; Roche; Merck KGaA). Western blot analysis was performed according to previously described methods (Park et al., 2024). The blots were analyzed using a chemiluminescence detector. Details of the primary antibodies used are listed in Table 1.

**TABLE 1:** List of antibodies for Western blot analyses.

Antigen	Host	Clonality	Manufacturer	Catalog number
FGF2	Rabbit	Polyclonal	Santa Cruz	sc-79
FGF7	Mouse	Monoclonal	Santa Cruz	sc-365440
IGF-1	Mouse	Monoclonal	Santa Cruz	sc-74116
NOG	Mouse	Monoclonal	Santa Cruz	sc-293439
VEGF	Mouse	Monoclonal	Santa Cruz	sc-7269
FGF10	Rabbit	Polyclonal	Millipore	ABN44
Cyclin B1	Mouse	Monoclonal	Santa Cruz	sc-245
Ki67	Rabbit	Polyclonal	Abcam	ab15580
K15	Rabbit	Monoclonal	Abcam	ab52816
K19	Mouse	Monoclonal	Invitrogen	MA5-12663
ANGPTL4	Mouse	Monoclonal	Santa Cruz	sc-373761
CD34	Rabbit	Monoclonal	Abcam	ab81289
p63α	Rabbit	Monoclonal	Cell Signaling Tech	13109
PPAR-α	Mouse	Monoclonal	Santa Cruz	sc-398394
PPAR-δ	Rabbit	Polyclonal	Santa Cruz	sc-7197
PPAR-γ	Mouse	Monoclonal	Santa Cruz	sc-7273
SAPK/JNK	Rabbit	Polyclonal	Cell Signaling Tech	9252
phospho-SAPK/JNK	Rabbit	Polyclonal	Cell Signaling Tech	9251
p38	Rabbit	Monoclonal	Cell Signaling Tech	54470
phospho-p38	Rabbit	Polyclonal	Cell Signaling Tech	9211
ERK	Rabbit	Polyclonal	Cell Signaling Tech	9102
phospho-ERK	Rabbit	Polyclonal	Cell Signaling Tech	9101
PKA	Rabbit	Polyclonal	Cell Signaling Tech	4782
phospho-PKA	Rabbit	Polyclonal	Cell Signaling Tech	4781
MAPKAPK2	Rabbit	Monoclonal	Cell Signaling Tech	12155
phospho-MAPKAPK2	Rabbit	Monoclonal	Cell Signaling Tech	3007
α-Tubulin	Mouse	Monoclonal	Cell Signaling Tech	sc-8035
Lamin A/C	Mouse	Monoclonal	Santa Cruz	4777
β-Actin	Mouse	Monoclonal	Santa Cruz	sc-47778

### 2.11 Enzyme-linked immunosorbent assay

HHFSCs (1 × 10⁵) and HDPCs (1.5 × 10⁵) were seeded in 60 mm culture dishes and incubated at 37°C for 24 h. Following incubation, the cells were treated with araliadiol at concentrations ranging from 0 to 2.5 μg/mL, either in the presence or absence of SB202190 (20 µM) or GW9662 (20 µM), for 48 h at 37°C. Minoxidil (2 μg/mL) was included as a positive control. After the treatment, the culture medium was centrifuged at 2,000 × g for 10 min, and the resulting supernatant was filtered through a 0.2 µm syringe filter (#S6534; Sartorius, Germany). The VEGF concentration in the conditioned medium was then quantified using a VEGF-A ELISA kit (#BMS277-2; Invitrogen; Thermo Fisher Scientific), following the manufacturer’s instructions.

### 2.12 Statistical analysis

In this study, statistical analyses were performed on a minimum of three independent experiments. One-way analysis of variance (ANOVA) was utilized to assess the statistical differences among the groups using GraphPad Prism software (version 8.0.1; CA, United States). When statistical significance was identified, Tukey’s test was applied to compare the means of multiple groups within each treatment category. The data are expressed as mean values ±standard deviation (SD), and the significance between groups was determined with a *p*-value threshold of less than 0.05.

## 3 Results

### 3.1 Identification and chromatographic analysis of araliadiol

**FIGURE 1:**
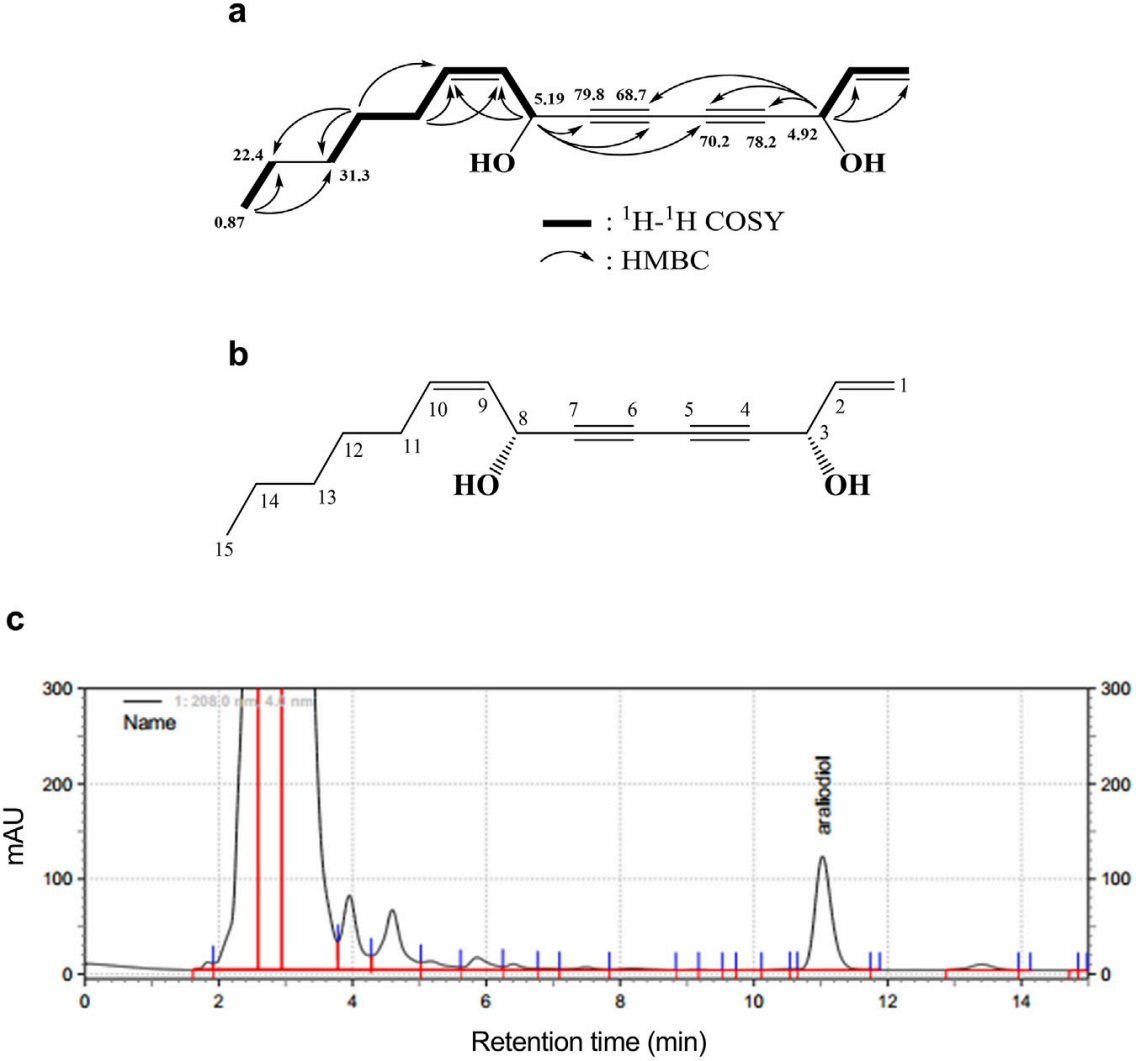
Identification and chromatographic analysis of araliadiol. **(A)** ^1^H–^1^H COSY and HMBC correlations of the isolated compound from *Centella asiatica* extract. **(B)** Chemical structure of isolated compound, araliadiol. **(C)** HPLC chromatogram of *Centella asiatica* extract at 208 nm. *Centella asiatica*, *Centella asiatica*.

An active compound, araliadiol, was obtained from *C. asiatica* extract as a colorless oil and exhibited UV maxima at 232, 243, and 254 nm. Its molecular weight was determined as 232 through ESI mass measurement, which showed a quasi-molecular ion peak at 255.1 [M + Na]^+^. The ^1^H NMR spectrum of araliadiol measured in CDCl_3_ showed signals due to three olefinic methines at δ 5.92 (m), 5.60 (ddt, *J* = 11.5, 0.5, 7.5 Hz), and 5.51 (ddt, *J* = 11.5, 8.0, 1.5 Hz), one terminal methylene at δ 5.45 (d, *J* = 17.5 Hz)/δ 5.24 (d, *J* = 10.0 Hz), two oxygenated methines at δ 5.19 (d, *J* = 8.0 Hz) and δ 4.92 (d, *J* = 5.5 Hz), four methylenes at δ 2.09 (m), 1.37 (m), 1.28 (m), and 1.26 (m), and one methyl at δ 0.87 (t, *J* = 7.0 Hz). In the ^13^C NMR spectrum with aid from HMQC spectrum, three sp^2^ methine carbons at δ 135.7, 134.6, and 127.6, one terminal methylene carbon at δ 117.3, four quaternary carbons at δ 79.8, 78.2, 70.2, and 68.7, two oxygenated methine carbons at δ 63.4 and 58.5, four methylene carbons at δ 31.3, 28.9, 27.6, and 22.4, and one methyl carbon at δ 14.0 were evident (Table 2). As shown in Figure 1A, the ^1^H–^1^H COSY spectrum established three structural fragments and the HMQC spectrum assigned all proton-bearing carbons. The HMBC correlations from the methyl protons at δ 0.87 to methylene carbons at δ 31.3 and 22.4, from an oxygenated methine proton at δ 5.19 to two sp^2^ methine carbons at δ 134.6 and 127.6 and three quaternary carbons at δ 79.8, 70.2, and 68.7, and from an oxygenated methine proton at δ 4.92 to carbons at δ 135.7, 117.3, 78.2, 70.2, and 68.7 were evident (Figure 1A). The carbons at δ 79.8, 78.2, 70.2, and 68.7 were proposed as sp carbons on the basis of their chemical shifts and molecular weight. Thus, the structure of the active compound was determined as shown in Figure 1B. This compound was identified as araliadiol by comparing ^1^H and ^13^C NMR spectral data with those reported in the literature (Cheng et al., 2011). Also, HPLC analysis of *C. asiatica* extract showed that the highest peak was detected at 11.027 min (Figure 1C). Collectively, NMR spectral data and other experimental data of the isolated compound were in good agreement with those of araliadiol.

**TABLE 2:** ^1^H and^13^C NMR spectral data of the isolated compound in CDCl_3_^*a*^.

No.	δ_C_	δ_H_
1	117.3	5.45 (1H, d, *J* = 17.5)^*b*^ 5.24 (1H, d, *J* = 10.0)
2	135.7	5.92 (1H, m)
3	63.4	4.92 (1H, d, *J* = 5.5)
4	78.2	
5	70.2	
6	68.7	
7	79.8	
8	58.5	5.19 (1H, d, *J* = 8.0)
9	127.6	5.51 (1H, ddt, *J* = 11.5, 8.0, 1.5)
10	134.6	5.60 (1H, ddt, *J* = 11.5, 0.5, 7.5)
11	27.6	2.09 (2H, m)
12	28.9	1.37 (2H, m)
13	31.3	1.28 (2H, m)
14	22.4	1.26 (2H, m)
15	14.0	0.87 (3H, t, *J* = 7.0)

### 3.2 Screening the pharmacological potential of araliadiol to promote hair growth

**FIGURE 2:**
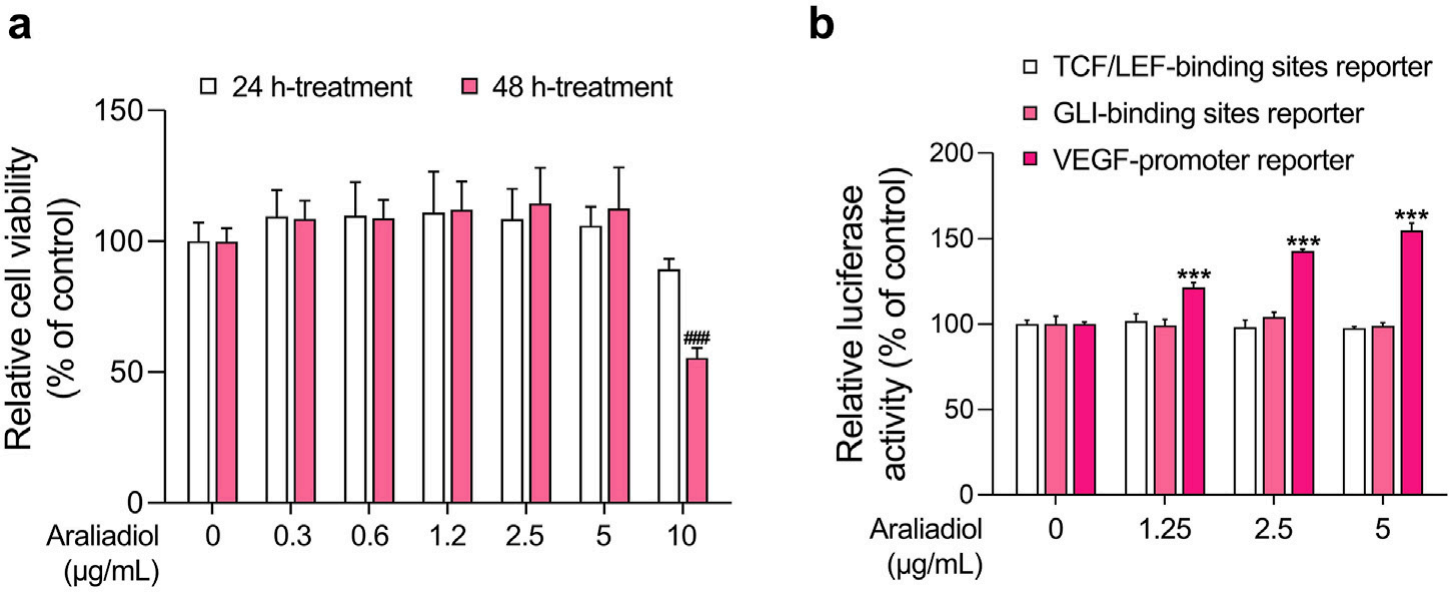
Effects of araliadiol on hair growth-related signaling pathways in 293T cells. **(A)** 293T cells were treated with varying concentrations of araliadiol (0–10 μg/mL) for up to 48 h, and cell viability was assessed using the WST-1 assay. **(B)** 293T cells were treated with araliadiol (0–5 μg/mL) for 48 h, and a luciferase reporter assay was conducted to evaluate the effects of araliadiol on hair growth-related signaling pathways. Luminescence values were normalized to β-galactosidase activities. Results are presented as the mean ± SD of three independent experiments and analyzed using a one-way analysis of variance followed by Tukey’s test. ^###, ***^ *p* < 0.001 compared with the vehicle-treated group. WST-1, water soluble tetrazolium salt 1.

The hair follicle is a highly complex skin appendage composed of different cell populations and undergoes a unique cyclic process called the hair cycle (Schneider et al., 2009). The hair cycle consists of anagen (growth phase), catagen (apoptosis-driven regression phase), and telogen (relative quiescence phase). In a healthy state, the ratio of anagen to telogen is maintained at approximately 12:1. However, several factors can disrupt this balance, increasing the proportion of telogen to become similar to anagen and thereby leading to alopecia (Schneider et al., 2009; Natarelli et al., 2023). Therefore, recent efforts to treat hair loss have focused on increasing the telogen-to-anagen transition. Representative methods aimed at increasing anagen follicles include topical minoxidil application, platelet-rich plasma therapy, and low-level laser therapy (Avci et al., 2014; Stevens and Khetarpal, 2019; Feaster et al., 2023). From a cellular perspective, the activation of hair follicle stem cells (HFSCs) and dermal papilla cells (DPCs) is crucial for the transition from telogen to anagen (Hsu et al., 2011). In particular, the activation of these cells requires the enhancement of different signaling pathways. The representative anagen-inducing signals include Wingless-related integration site (WNT)/β-Catenin, Sonic hedgehog (Shh), and vascular endothelial growth factor (VEGF) signaling (Choi, 2020). Therefore, we investigated whether araliadiol enhances the three signaling pathways to evaluate its pharmacological potential in promoting hair growth and the anagen phase (Figure 2).

To this end, we conducted a luciferase-based reporter assay to assess the activation of these pathways. As shown in Figure 2, araliadiol did not induce the transcriptional activity of β-Catenin and Gli at non-cytotoxic concentrations (0–5 μg/mL). However, it increased the activity of VEGF promoter-driven luciferase in a dose-dependent manner. Specifically, the luciferase activity was increased by 121.54%, 142.66%, and 154.71%, respectively, in the groups treated with 1.25, 2.5, and 5 μg/mL of araliadiol, compared to the untreated group (Figure 2B). Therefore, these results suggest that araliadiol may possess pharmacological efficacy in promoting *VEGF* expression.

### 3.3 Araliadiol enhances proliferative capacities in human hair follicle cells

Although the transcriptional activity of β-Catenin and Gli is important for accelerating anagen hair cycle, the increase in VEGF expression is also well recognized as a key factor for inducing hair growth (Yano et al., 2001; Paladini et al., 2005; Ahmed et al., 2017). Having revealed the VEGF expression-enhancing effect of araliadiol in 293T cells, we next determined whether araliadiol exerts *in vitro* hair growth-promoting effects on primary cells constituting actual hair follicles. To this end, we first measured cytotoxicity using a WST-1 assay in HDPCs and HHFSCs.

**FIGURE 3:**
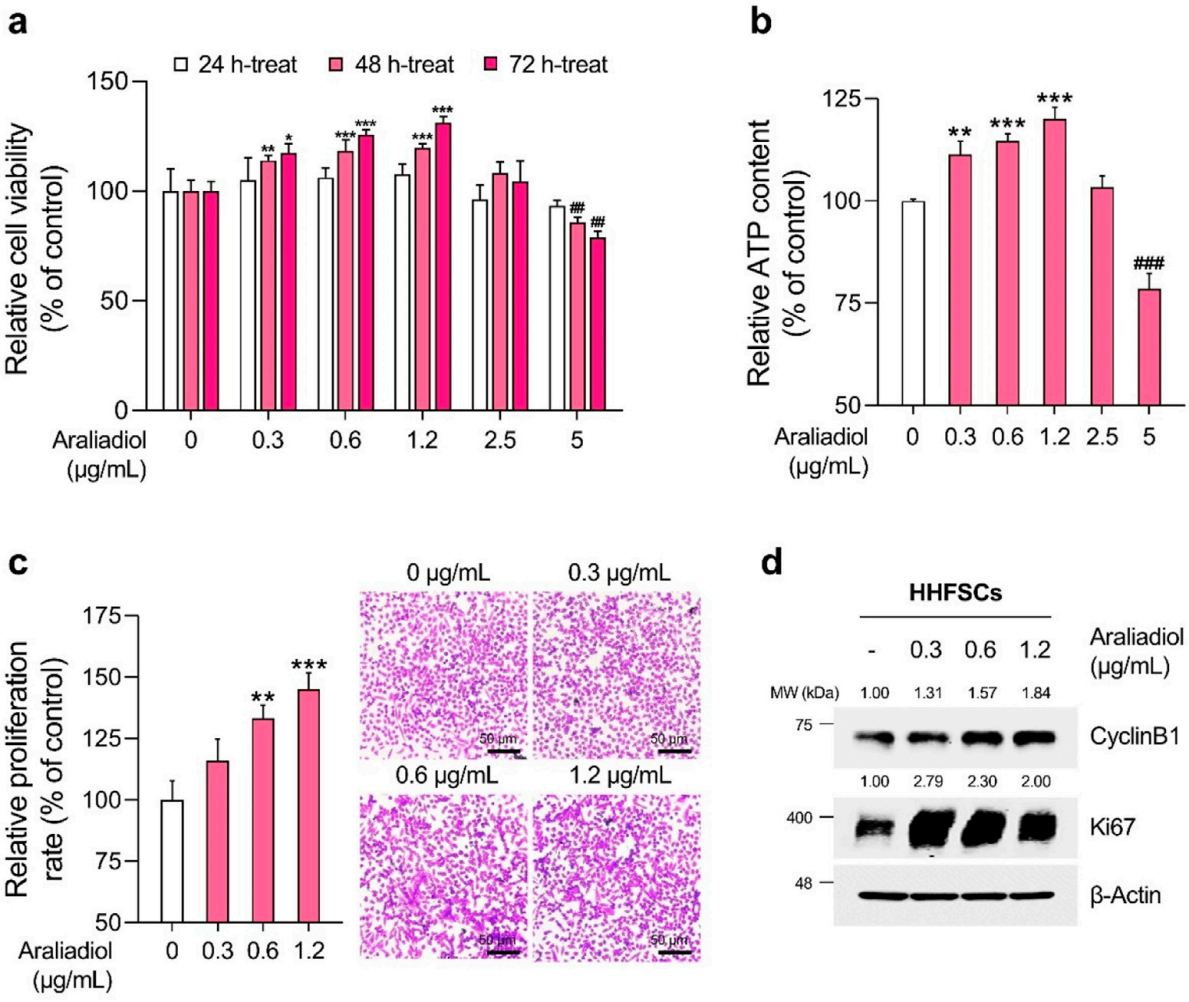
Araliadiol promotes proliferation in human hair follicle stem cells within non-toxic concentrations. **(A)** HHFSCs were treated with araliadiol (0–5 μg/mL) for up to 72 h, and cell viability was assessed using the WST-1 assay. **(B, C)** HHFSCs were treated with araliadiol (0–5 μg/mL) for 72 h, and their proliferative capacity was evaluated using an intracellular ATP detection assay **(B)** and crystal violet staining assay **(C, D)** Protein expression levels of cell cycle-related markers (CyclinB1 and Ki67) were analyzed by Western blotting and normalized to β-Actin levels. Results are presented as the mean ± SD of three independent experiments and analyzed using a one-way analysis of variance followed by Tukey’s test. ^*^ *p* < 0.05; ^##,**^ *p* < 0.01; ^###, ***^ *p* < 0.001 compared with the vehicle-treated group. HHFSCs, human hair follicle stem cells; ATP, adenosine triphosphate.

**FIGURE 4:**
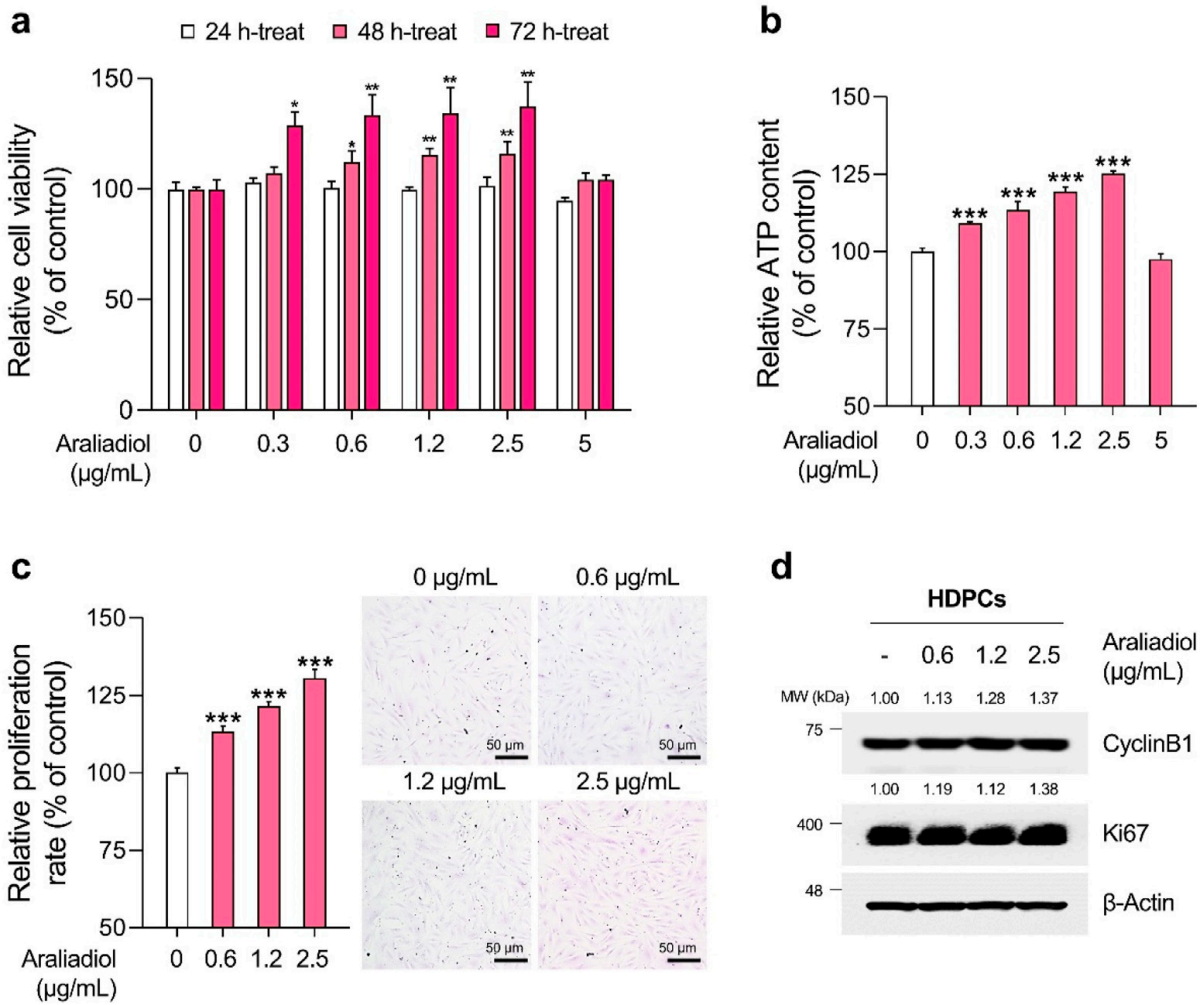
Araliadiol promotes proliferation in human dermal papilla cells within non-toxic concentrations. **(A)** HDPCs were treated with araliadiol (0–5 μg/mL) for up to 72 h, and cell viability was assessed using the WST-1 assay. **(B, C)** HDPCs were treated with araliadiol (0–5 μg/mL) for 72 h, and their proliferative capacity was evaluated using an intracellular ATP detection assay **(B)** and crystal violet staining assay **(C, D)** Protein expression levels of cell cycle-related markers (CyclinB1 and Ki67) were analyzed by Western blotting and normalized to β-Actin levels. Results are presented as the mean ± SD of three independent experiments and analyzed using a one-way analysis of variance followed by Tukey’s test. ^*^ *p* < 0.05; ^**^ *p* < 0.01; ^***^ *p* < 0.001 compared with the vehicle-treated group. HDPCs, human hair follicle dermal papilla cells.

As shown in Figures 3A, 4A, araliadiol did not exhibit significant toxicity in HHFSCs and HDPCs at concentrations up to 2.5 μg/mL and 5 μg/mL, respectively. Although araliadiol exhibited cytotoxicity at higher concentrations in both cell types (IC_50_ = 13.750 μg/mL for HHFSCs and 21.831 μg/mL for HDPCs), it demonstrated a proliferative effect at lower concentrations (Supplementary Figure S2). Specifically, in the low concentration range of araliadiol (up to 2.5 μg/mL), a mild proliferative effect was observed in both cell types. Cell viability increased to 131.09% in HHFSCs treated with 1.2 μg/mL of araliadiol and to 137.43% in HDPCs treated with 2.5 μg/mL of araliadiol for 72 h. The proliferation of cells constituting hair follicles is recognized as a major hallmark of anagen cycling. Indeed, promoting the proliferation of HHFSCs and HDPCs using different chemicals can induce hair follicle regeneration (Han et al., 2004; Krause and Foitzik, 2006; Qiu et al., 2017; Kim et al., 2019). The WST-1 assay, used to measure cytotoxicity, is a tetrazolium reduction-based method for evaluating intracellular metabolic activity (Krause and Foitzik, 2006). Therefore, we validated the proliferative effects of araliadiol on hair follicle cells through additional biochemical analyses.

As depicted in Figures 3B, 4B, the ATP content in HHFSCs and HDPCs treated with araliadiol for 72 h increased to a maximum of 120.01% and 125.17%, respectively. Specifically, the intracellular ATP levels increased in a dose-dependent manner, similar to the results shown in Figures 3A, 4A. In addition, the proliferative effects of araliadiol on hair follicle cells were confirmed through crystal violet staining. Crystal violet is a triarylmethane dye that binds to intracellular DNA, staining only live cells adhered to the plate. Consequently, this method is widely used in several studies to analyze cell proliferation rates (Feoktistova et al., 2016). As shown in Figure 3C, the cell proliferation rate increased to 115.94%, 133.20%, and 145.18%, respectively, following the treatment of HHFSCs with 0.3, 0.6, and 1.2 μg/mL of araliadiol. Similarly, in HDPCs treated with araliadiol for 72 h, the cell proliferation rate increased up to 130.59%, further confirming the proliferative effects of araliadiol (Figure 4C). Next, we performed a Western blot analysis to examine cell cycle progression in hair follicle cells treated with araliadiol. We analyzed the protein levels of Ki67 and Cyclin B1, cell cycle markers known to directly influence mitosis. As shown in Figures 3D, 4D, the protein expression of Cyclin B1 and Ki67 was elevated in a dose-dependent manner in hair follicle cells treated with araliadiol. Collectively, these results indicate that araliadiol can mildly promote mitotic cell division in human hair follicle cells.

### 3.4 Araliadiol accelerates hair growth-promoting signals in human hair follicle cells

The results from Figures 2–4 demonstrate that araliadiol exerts a highly positive impact on hair growth at the cellular level. Therefore, in subsequent experiments, we determined whether araliadiol can enhance hair growth-promoting and anagen-inductive signals in two types of human hair follicle cells: HHFSCs and HDPCs.

**FIGURE 5:**
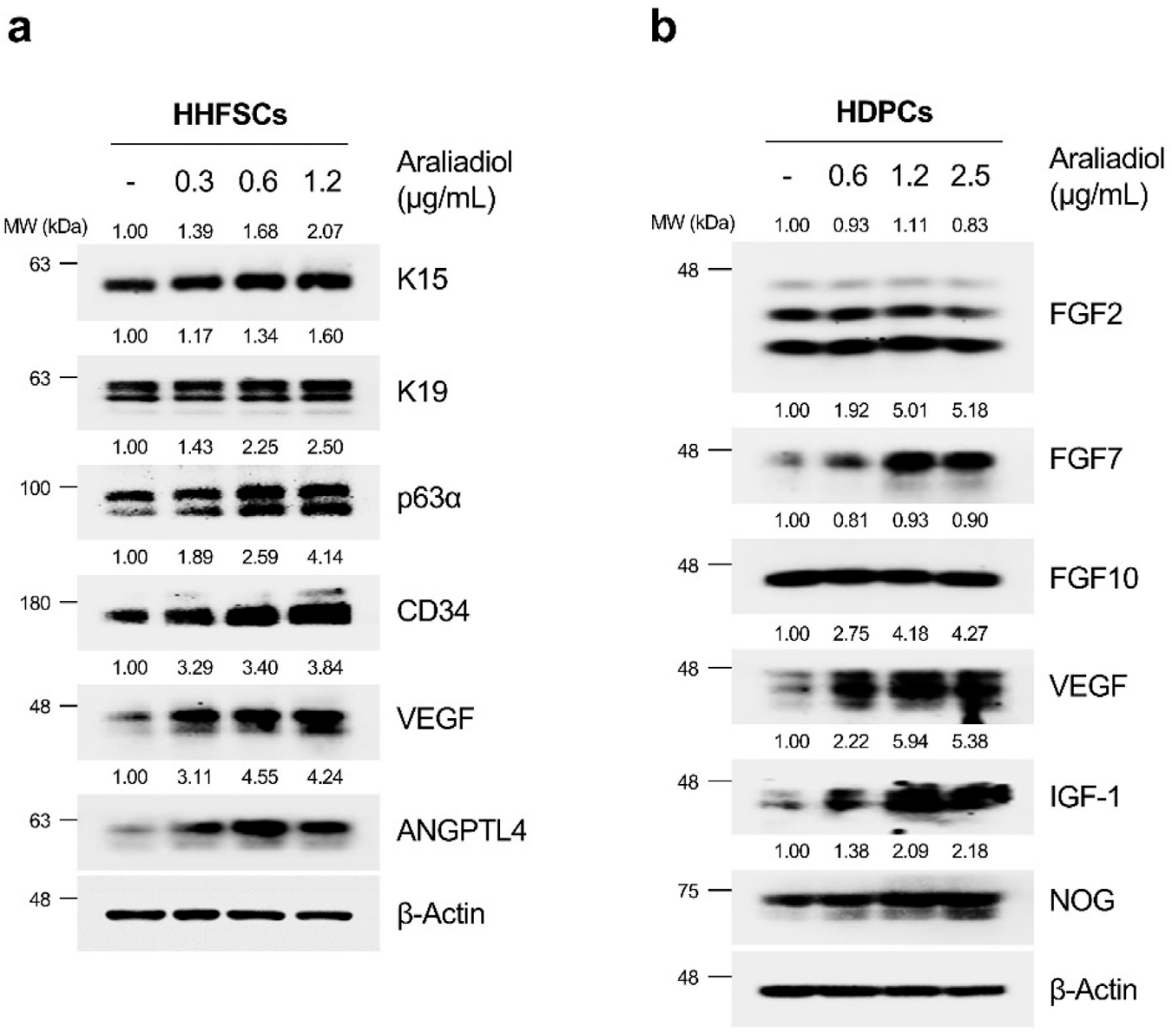
Araliadiol enhances hair growth-promoting signals and anagen phase markers in human hair follicle cells. **(A)** HHFSCs were treated with different concentrations of araliadiol (0–1.2 μg/mL) for 48 h. Protein levels of hair follicle stem cell-specific markers (K15 and K19), anagen phase markers (CD34 and p63α), and hair growth-promoting proteins (VEGF and ANGPTL4) were analyzed by Western blotting, with β-Actin serving as a loading control. **(B)** HDPCs were stimulated with indicated concentrations of araliadiol (0–2.5 μg/mL) for 48 h. Protein levels of hair growth-promoting proteins (FGF2, FGF7, FGF10, VEGF, IGF-1, and NOG) were analyzed by Western blotting, with β-Actin serving as a loading control. The protein levels in all blots were quantified using ImageJ software version 1.53t. K15, cytokeratin 15; K19, cytokeratin 19; CD34, cluster of differentiation 34; p63α, tumor protein 63 alpha; VEGF, vascular endothelial growth factor; ANGPTL4, angiopoietin like 4; FGF2, fibroblast growth factor 2; FGF7, fibroblast growth factor 7; FGF10, fibroblast growth factor 10; IGF-1, insulin-like growth factor 1; NOG, noggin.

The progression of the anagen follicle requires essential crosstalk between mesenchymal and epithelial lineage cells constituting the hair follicle (Rendl et al., 2005). In particular, several paracrine factors secreted by these cells are known to promote hair growth. For instance, in HHFSCs, angiogenic factors such as angiopoietin-like (ANGPTL) 4, ANGPTL7, and VEGF are well-known, whereas in HDPCs, VEGF, Noggin (NOG), insulin-like growth factor-1 (IGF-1), fibroblast growth factor (FGF) 7, FGF10, and FGF2 are recognized as secreted proteins that induce hair growth (Cazzola and Perez-Moreno, 2022; Kim and Sung, 2023). Therefore, we investigated how araliadiol affects the expression of anagen follicle markers and hair growth-promoting factors. As shown in Figure 5A, we observed an increase in the protein levels of HFSC-specific markers (K15 and K19), anagen phase markers (CD34 and p63α), and hair growth-promoting factors (VEGF and ANGPTL4) in araliadiol-treated HHFSCs. In addition, in HDPCs treated with araliadiol for 48 h, the protein expression of FGF7, VEGF, IGF-1, and NOG increased in a dose-dependent manner, whereas the levels of FGF2 and FGF10 remained unchanged (Figure 5B). These results demonstrate that araliadiol promotes different hair growth-promoting signals known to be important in HHFSCs and HDPCs, and induces an increase in intracellular biomarkers associated with the hair follicle anagen state.

### 3.5 Araliadiol induces transcriptional activity and protein stability of PPAR-γ in human hair follicle cells

The results shown in Figure 5 indicate that araliadiol upregulates hair growth signals in HHFSCs and HDPCs. Consequently, we investigated the transcription factors activated by araliadiol to enhance hair growth signaling. Specifically, K15 and K19, known markers of hair follicle stem cells, and VEGF and ANGPTL4, known to induce the anagen phase in HFSCs, are well-established downstream targets of PPAR-γ (Gealekman et al., 2008; Ramot et al., 2020). Similarly, VEGF, FGF7, and IGF-1, which appear to be increased in HDPCs, are also known to be regulated by PPAR-γ. As shown in Figure 2B, araliadiol does not regulate β-Catenin or Gli signaling. Therefore, we next verified the potential of PPAR-γ as a regulator of VEGF, a key factor in hair growth induction.

**FIGURE 6:**
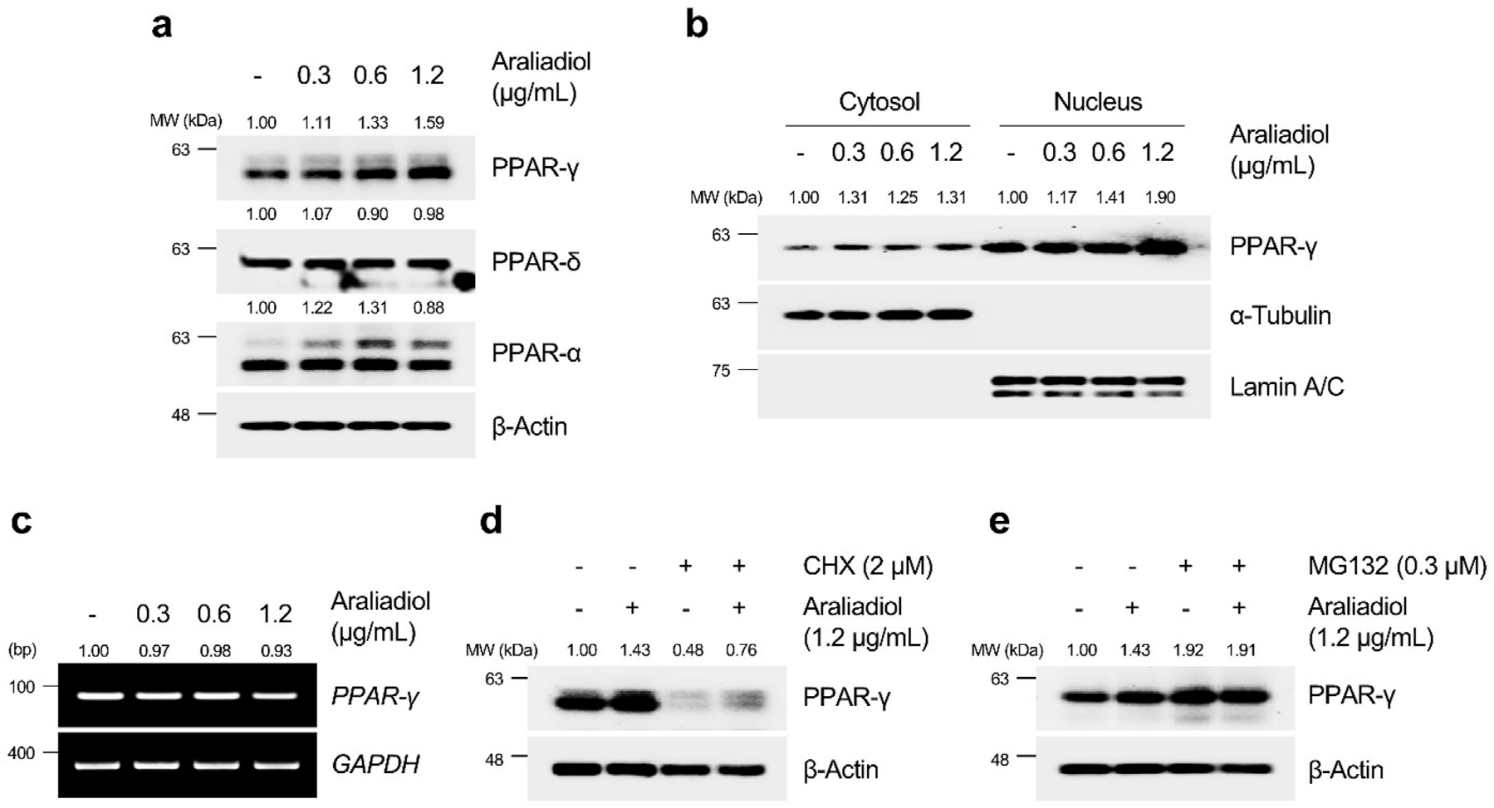
Araliadiol induces transcriptional activity of PPAR-γ by elevating protein stability rather than increasing mRNA expression in HHFSCs. **(A)** HHFSCs were treated with different concentrations of araliadiol (0–1.2 μg/mL) for 48 h. Protein levels of the PPAR family (PPAR-α, PPAR-δ, and PPAR-γ) were analyzed by Western blotting, with β-Actin as a loading control. **(B)** Nuclear translocation of PPAR-γ was analyzed by Western blotting. α-Tubulin and Lamin A/C served as loading controls for cytoplasmic and nuclear fractions, respectively. **(C)** mRNA expression of *PPAR-γ* was determined by PCR and normalized to *GAPDH*. **(D)** Protein stability of PPAR-γ was assessed after treatment with araliadiol (1.2 μg/mL) with or without CHX (2 μM) in HHFSCs for 48 h **(E)** HHFSCs were treated with araliadiol (0–1.2 μg/mL) with or without MG132 (0.3 μM) for 48 h, and protein levels of PPAR-γ were analyzed by Western blotting with β-actin as a loading control. mRNA and protein levels in all blots were quantified using ImageJ software version 1.53t. PCR, polymerase chain reaction; PPAR, peroxisome proliferator-activated receptor; GAPDH, glyceraldehyde 3-phosphate dehydrogenase.

**FIGURE 7:**
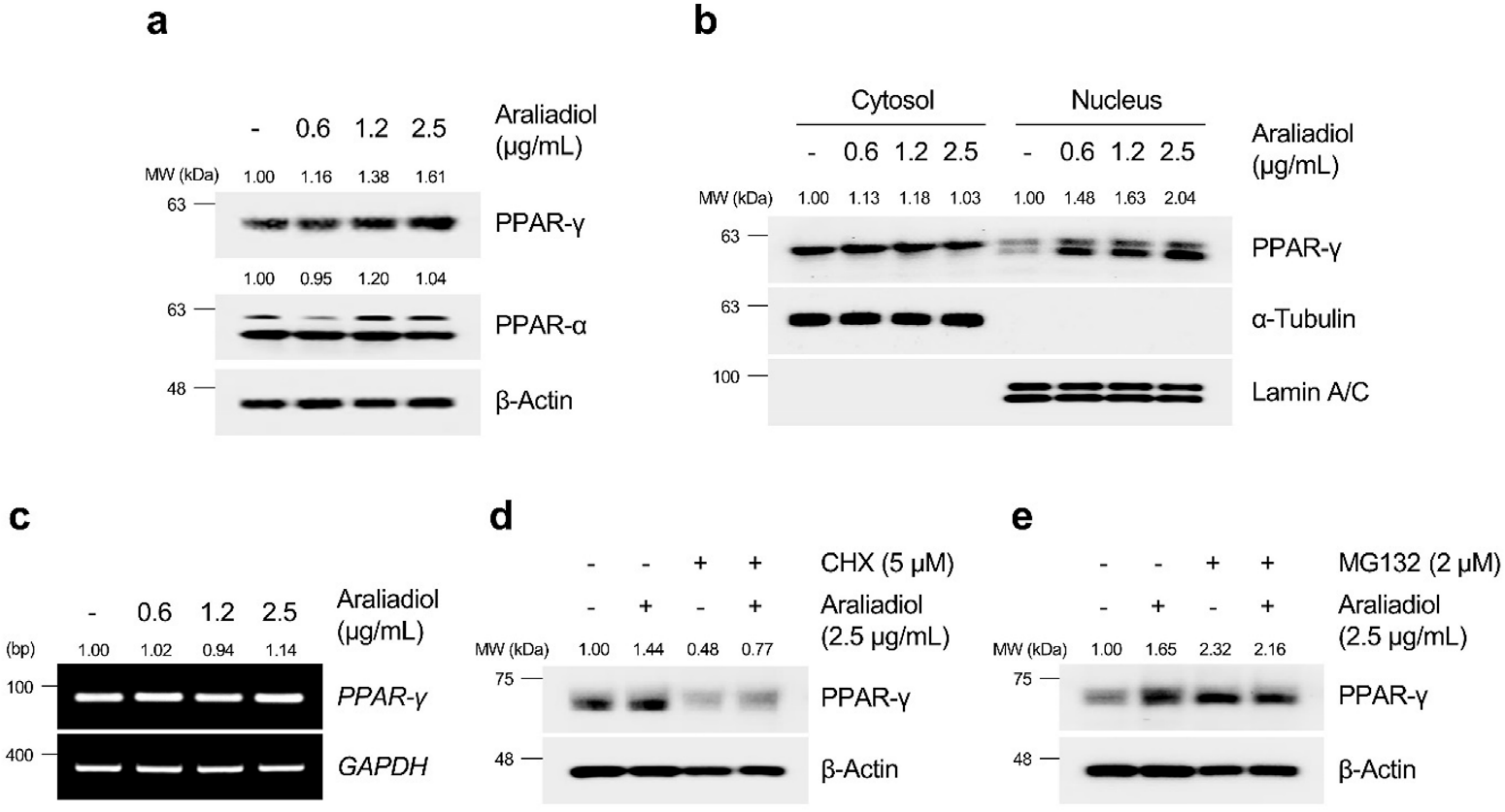
Araliadiol induces transcriptional activity of PPAR-γ by elevating protein stability rather than increasing mRNA expression in HDPCs. **(A)** HDPCs were treated with different concentrations of araliadiol (0–2.5 μg/mL) for 48 h. Protein levels of PPAR-α and PPAR-γ were analyzed by Western blotting with β-Actin as a loading control. **(B)** Nuclear translocation of PPAR-γ was analyzed by Western blotting. α-Tubulin and Lamin A/C served as loading controls for the cytoplasmic and nuclear fractions, respectively. **(C)** mRNA expression of *PPAR-γ* was determined by PCR and normalized to *GAPDH*. **(D)** Protein stability of PPAR-γ was assessed after treatment with araliadiol (2.5 μg/mL) with or without CHX (5 μM) in HDPCs for 48 h **(E)** HDPCs were treated with araliadiol (0–2.5 μg/mL) with or without MG132 (2 μM) for 48 h, and protein levels of PPAR-γ were analyzed by Western blotting, with β-Actin as a loading control. mRNA and protein levels in all blots were quantified using ImageJ software version 1.53t.

As shown in Figures 6A, 7A, araliadiol significantly increased the protein levels of PPAR-γ compared to those of PPAR-δ and PPAR-α in hair follicle cells. Because PPAR-γ functions as a transcription factor, an increase in protein expression does not always correlate with transcriptional activation. Thus, we performed a cell fractionation assay to separate the cytoplasmic and nuclear parts of hair follicle cells to confirm the protein expression of PPAR-γ in the nucleus (Figures 6B, 7B). The results demonstrated that araliadiol treatment increased the protein levels of PPAR-γ in both the cytoplasm and nucleus of HDPCs and HHFSCs. This finding suggests that PPAR-γ undergoes posttranslational modifications, causing increased protein stability. To determine whether the increased protein expression of PPAR-γ was due to elevated transcription or posttranscriptional modification, we measured mRNA levels. Our results demonstrated that the mRNA expression of PPAR-γ did not change in hair follicle cells treated with araliadiol (Figures 6C, 7C). However, when co-treated with cycloheximide, a known inhibitor of eukaryotic translation, further increased the protein level of PPAR-γ compared to cycloheximide treatment alone (Figures 6D, 7D). These results suggest that araliadiol enhances protein expression by increasing the stability of PPAR-γ protein in human hair follicle cells.

The ubiquitin–proteasome system (UPS) is a well-known pathway that regulates protein homeostasis in cells (Nandi et al., 2006). For intracellular protein homeostasis, proteasomal degradation is necessary, where several proteins are ubiquitinated by E3 ligase, marking them for degradation by the proteasome (Eldridge and O’brien, 2010). It has been reported that PPAR-γ, which performs different physiological roles in cells, can also undergo proteasome-dependent degradation through polyubiquitin conjugation (Floyd and Stephens, 2002). Therefore, we investigated whether araliadiol increases protein stability through proteasome inhibition rather than by increasing mRNA expression. As shown in Figures 6E, 7E, PPAR-γ expression increased in cells treated with MG132 alone, and co-treatment with MG132 and araliadiol did not further increase the protein expression compared to MG132 treatment alone. These results suggest that araliadiol increases protein expression and transcriptional activity by inhibiting the proteasomal degradation of PPAR-γ. Collectively, our results indicate that araliadiol enhances hair growth signals by increasing the transcriptional activity of PPAR-γ through the inhibition of the proteasomal degradation pathway, rather than increasing its mRNA expression.

### 3.6 Hair growth-promoting signals enhanced by araliadiol are regulated through the transcriptional activity of PPAR-γ

In Figures 6, 7, we confirmed that araliadiol enhances the protein stability and transcriptional activity of PPAR-γ by preventing its proteasomal degradation. To further investigate whether araliadiol’s upregulation of hair growth-promoting factors (e.g., VEGF) is regulated in a PPAR-γ- dependent manner, we conducted additional experiments.

**FIGURE 8:**
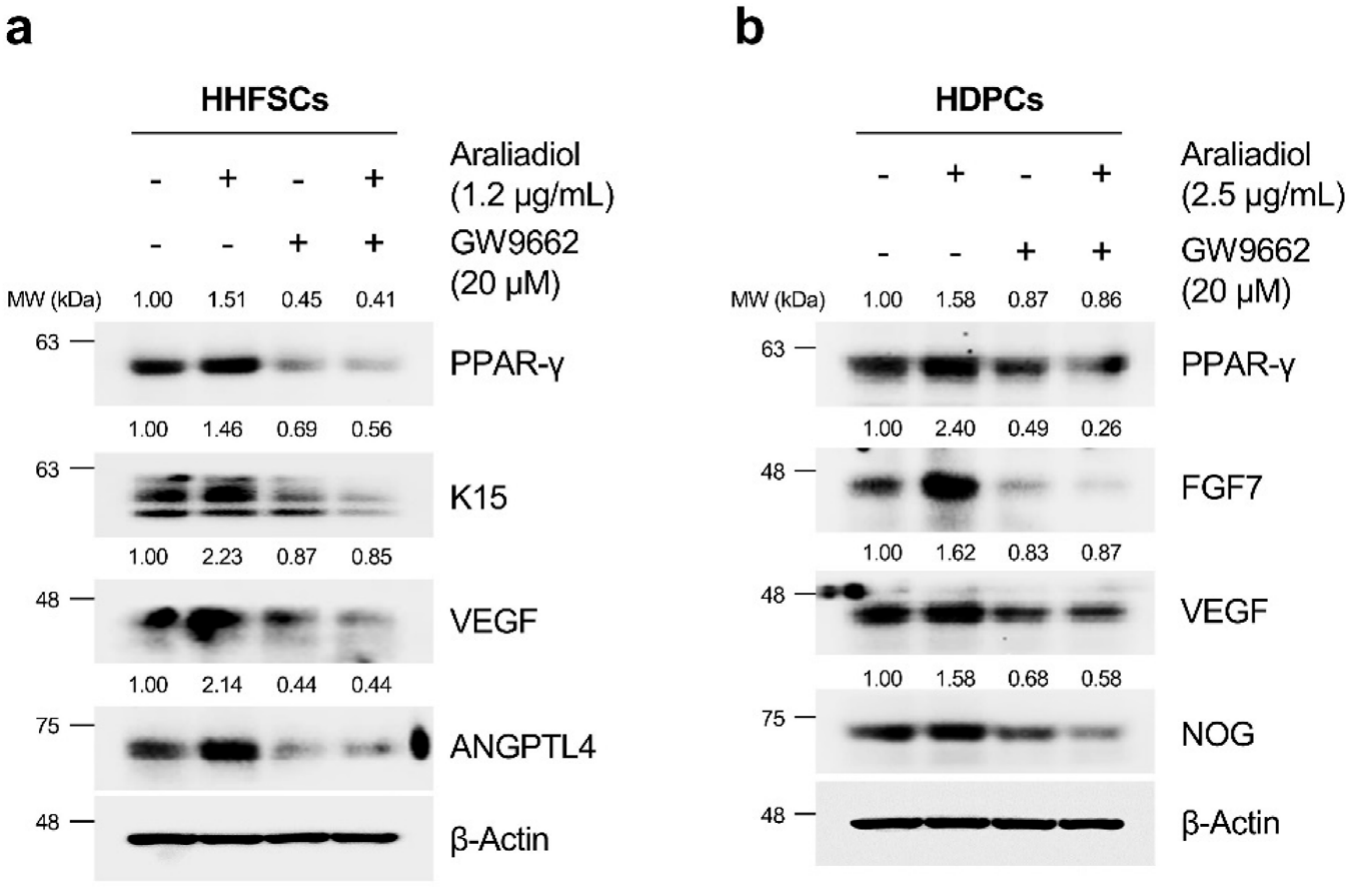
Araliadiol elevates hair growth-promoting effects in human hair follicle cells by upregulating the PPAR-γ signaling pathway. **(A)** Protein levels of PPAR-γ and hair growth-inductive proteins (K15, VEGF, and ANGPTL4) were assessed after treatment with araliadiol (1.2 μg/mL) with or without GW9662 (20 μM) in HHFSCs for 48 h. **(B)** Protein levels of PPAR-γ and hair growth-inductive proteins (FGF7, VEGF, and NOG) were assessed after treatment with araliadiol (2.5 μg/mL) with or without GW9662 (20 μM) in HDPCs for 48 h. Protein levels in all blots were quantified using ImageJ software version 1.53t.

As shown in Figure 8, araliadiol upregulated hair growth-promoting signals in both HHFSCs and HDPCs through PPAR-γ. Specifically, in HHFSCs treated with araliadiol alone, the expression of K15, VEGF, and ANGPTL4 increased. In contrast, in the negative control group treated with GW9662 alone, the protein levels of K15, VEGF, and ANGPTL4 were reduced compared to the untreated control. In addition, the levels of hair growth-inductive factors (K15, VEGF, ANGPTL4) enhanced by araliadiol were reduced to those similar to the negative control in cells co-treated with GW9662. Similarly, in HDPCs treated with araliadiol alone, the protein levels of FGF7, VEGF, and NOG increased. However, in cells co-treated with GW9662, these levels were reduced to match those in the negative control. These results suggest that araliadiol induces hair growth signaling through the PPAR-γ signaling in human hair follicle cells.

### 3.7 *In vitro* hair growth-promoting effects of araliadiol are dependent on p38/PPAR-γ signaling pathway in human hair follicle cells

Next, we elucidated the upstream signaling pathway through which araliadiol regulates the protein stability of PPAR-γ. PPAR-γ is known to undergo posttranslational modifications by different upstream kinases. Notably, kinases such as extracellular signal-regulated kinase (ERK), p38 mitogen-activated protein kinases (p38), c-Jun N-terminal kinase (JNK), and protein kinase A (PKA) are involved; ERK and JNK downregulate PPAR-γ′s transcriptional activity, whereas PKA and p38 upregulate it (Camp et al., 1999; Diradourian et al., 2005; Schild et al., 2006; Ptasinska et al., 2007; Kaplan et al., 2010). Therefore, we sought to determine which proteins regulate the stability and transcriptional activity of PPAR-γ in araliadiol-treated hair follicle cells.

**FIGURE 9:**
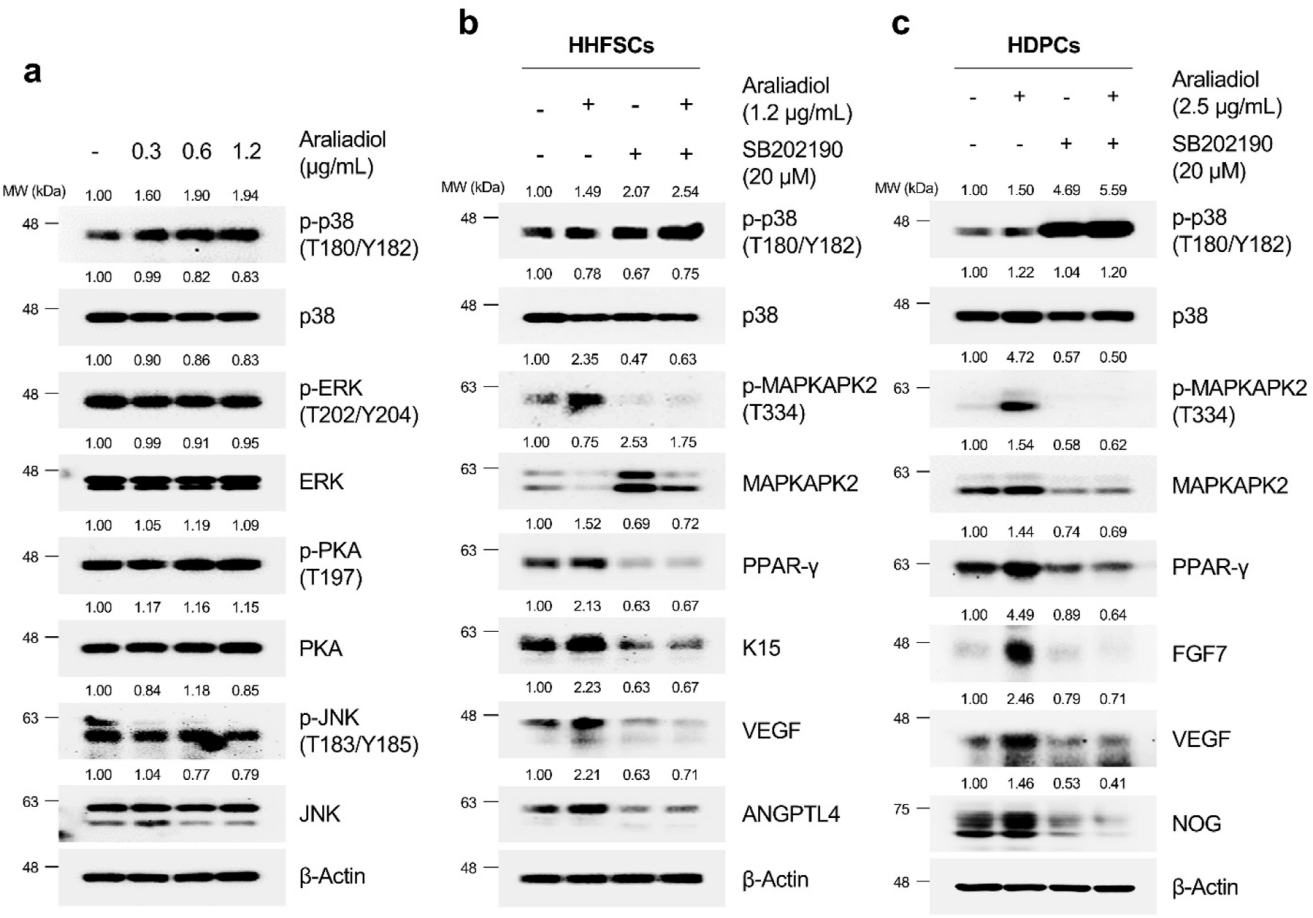
Araliadiol induces PPAR-γ/hair growth-promoting signaling by activating p38MAPK in human hair follicle cells. **(A)** Protein levels of PPAR-γ and its upstream kinases (p38, ERK, JNK, and PKA) were assessed after treatment with araliadiol (0–1.2 μg/mL) in HHFSCs for 48 h. The protein levels of the p38/PPAR-γ/hair growth-promoting signaling pathway were assessed after treatment with araliadiol with or without SB202190 (20 μM) in **(B)** HHFSCs and **(C)** HDPCs for 48 h. Protein levels in all blots were quantified using ImageJ software version 1.53t. p38, p38 mitogen-activated protein kinase; ERK, extracellular signal-regulated kinase; JNK, Jun N-terminal kinase; PKA, protein kinase **(A)**.

As shown in Figure 9A, in HHFSCs treated with araliadiol for 48 h, the phosphorylation levels of ERK, PKA, and JNK did not show significant changes; however, the phosphorylation level of p38 at T180/Y182 residues increased in a dose-dependent manner. Consequently, we confirmed whether the upregulation of PPAR-γ and hair growth-promoting signals by araliadiol is dependent on p38 signaling. We used SB202190 as the p38 inhibitor that inhibits the catalytic activity of p38 MAPK through ATP competition without suppressing the phosphorylation of p38 itself (Li et al., 2022). Hence, we assessed the phosphorylation level of its direct downstream target, MAPKAPK-2, to verify the catalytic activity of p38.

As shown in Figures 9B, C, treatment with araliadiol increased the phosphorylation levels of p38 and MAPKAPK2 in both HHFSCs and HDPCs. Conversely, in cells treated with SB202190 alone, the phosphorylation level of p38 was not reduced; however, the phosphorylation level of MAPKAPK-2 was significantly decreased. This finding is consistent with that of previous studies using SB202190 (Camp et al., 1999; Xu et al., 2006; Li et al., 2022). In addition, the inhibition of p38’s catalytic activity with SB202190 suppressed PPAR-γ and hair growth-promoting signals enhanced by araliadiol. Specifically, in HHFSCs treated with araliadiol alone, the protein expression of PPAR-γ, K15, VEGF, and ANGPTL4 increased, whereas treatment with SB202190 alone significantly decreased the expression of PPAR-γ, K15, VEGF, and ANGPTL4 compared to the control. Furthermore, in cells co-treated with araliadiol and SB202190, the enhanced PPAR-γ and hair growth-inductive proteins were downregulated to levels similar to those in the negative control, demonstrating that araliadiol regulates PPAR-γ and hair growth-promoting signals in human hair follicle cells in a p38 signaling-dependent manner. This trend was similarly observed in HDPCs, where treatment with araliadiol alone significantly increased the phosphorylation levels of p38 and MAPKAPK2, whereas co-treatment with SB202190 reduced the phosphorylation levels of MAPKAPK-2 to levels similar to the negative control. In addition, the increased protein levels of PPAR-γ, FGF7, and Noggin induced by araliadiol were significantly reduced upon co-treatment with SB202190.

Since many hair growth-promoting signals are secreted proteins, we next examined whether araliadiol enhances the secretion of proteins that promote the anagen phase in hair follicle cells. The transition from telogen to anagen phase, necessary for increasing hair follicle density and promoting hair growth, requires upregulation of key secreted markers. These include FGF7, FGF10, Noggin, and VEGF in HDPCs, and VEGF and ANGPTL4 in HHFSCs (Cazzola and Perez-Moreno, 2022; Kim and Sung, 2023). Among these, VEGF is the most prominent hair growth-promoting factor. Overexpression of VEGF in transgenic mice stimulates hair growth and angiogenesis while neutralizing VEGF attenuates these effects (Yano et al., 2001). *In vivo* studies further support the role of VEGF in promoting follicle growth, density, and angiogenesis (Ozeki and Tabata, 2002; Gnann et al., 2013), with decreased VEGF expression reported in patients with alopecia (Kubanov et al., 2017a; Kubanov et al., 2017b; El-Refai et al., 2020). These findings underscore VEGF’s crucial role in hair regeneration and growth.

**FIGURE 10:**
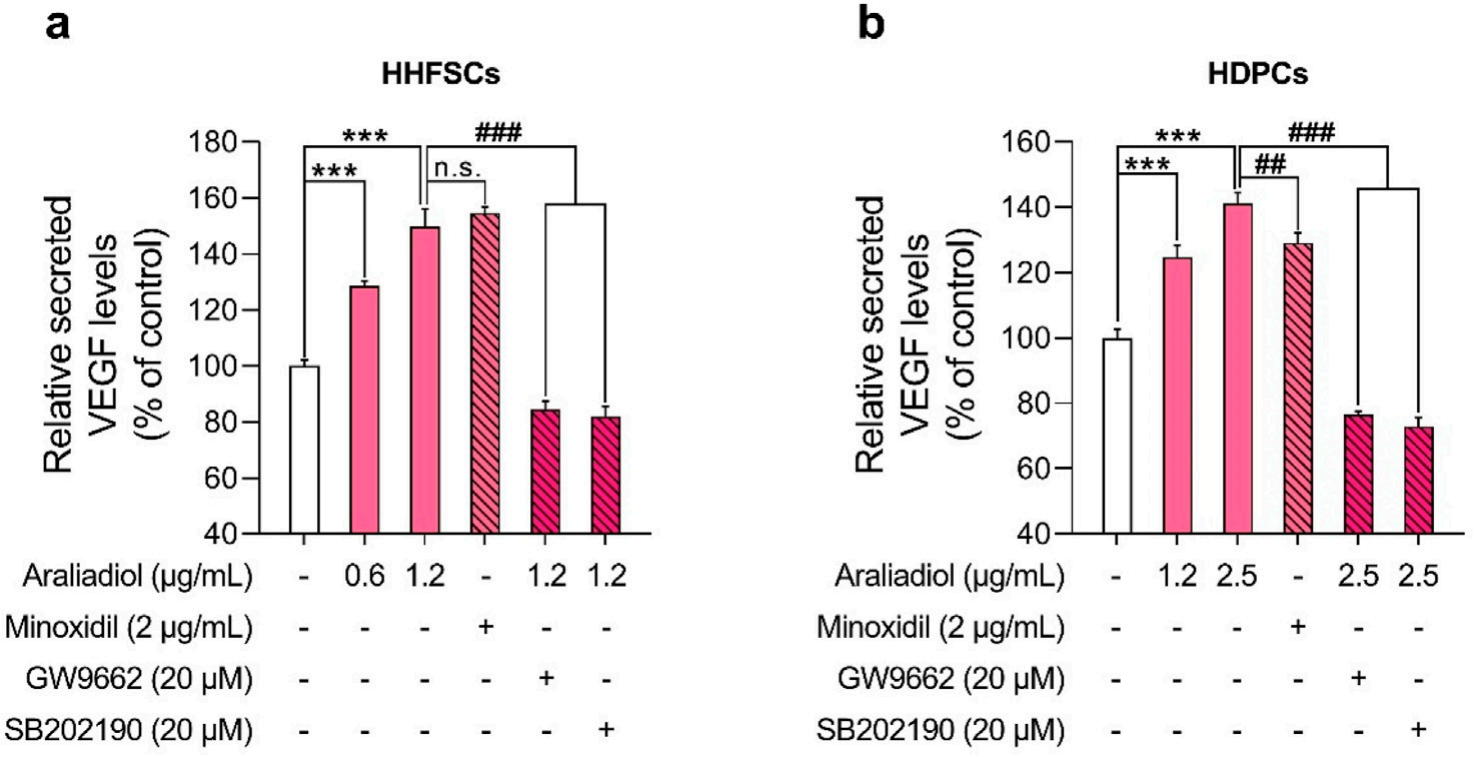
Araliadiol enhances VEGF secretion via the p38/PPAR-γ signaling pathway in human hair follicle cells. **(A, B)** Human hair follicle cells were treated with araliadiol (0–2.5 μg/mL) for 48 h, either with or without SB202190 (20 μM) or GW9662 (20 μM). VEGF-A secretion levels were quantified using an ELISA on conditioned medium from the treated cells. Minoxidil (2 μg/mL) was included as a positive control. Results are presented as the mean ± SD of three independent experiments and analyzed using a one-way analysis of variance followed by Tukey’s test. ^***^ *p* < 0.001 compared with the vehicle-treated group. ^##^ *p* < 0.01; ^###^ *p* < 0.001 compared with the araliadiol-treated group. ELISA, enzyme-linked immunosorbent assay.

In our study, araliadiol increased VEGF protein expression in a dose-dependent manner in both HDPCs and HHFSCs (Figure 5). To determine whether this increase correlated with enhanced VEGF secretion, we conducted an ELISA assay (Figure 10). In HHFSCs, treatment with 0.6 and 1.2 μg/mL araliadiol for 48 h resulted in VEGF secretion increasing by 128.65% and 149.91%, respectively (Figure 10A). Similarly, in HDPCs, araliadiol treatment at concentrations of 1.2 and 2.5 μg/mL led to VEGF secretion increases of 124.81% and 141.18%, respectively (Figure 10B). Minoxidil, used as a positive control, increased VEGF secretion by 154.58% in HHFSCs and 129.20% in HDPCs after 48 h of treatment, consistent with previous studies (Lachgar et al., 1998; Marubayashi et al., 2001). Compared to the minoxidil-treated group, VEGF levels in HHFSCs treated with 1.2 μg/mL araliadiol showed no significant difference, while in HDPCs treated with 2.5 μg/mL araliadiol, VEGF levels showed a slight increase (11.98%). Although minoxidil primarily acts as an ATP-sensitive potassium channel opener to induce vasodilation and increase blood flow, its hair growth-promoting effects are mediated by increased synthesis of prostaglandin E2 and VEGF (Messenger and Rundegren, 2004; Nagai et al., 2019). These findings suggest that araliadiol, producing VEGF increases comparable to those of minoxidil, shows potential as an effective treatment for hair loss.

To further assess whether VEGF secretion is mediated by the p38 and PPAR-γ signaling pathways, we co-treated cells with GW9662 (20 μM) or SB202190 (20 μM) alongside araliadiol. In both cell types, co-treatment with GW9662 or SB202190 significantly reduced VEGF secretion levels compared to treatment with araliadiol alone. Specifically, in HHFSCs, VEGF levels following treatment with 1.2 μg/mL araliadiol (149.91%) were reduced to 84.54% with GW9662 and to 82.01% with SB202190, both reductions being statistically significant (Figure 10A). Similarly, in HDPCs, VEGF levels following 2.5 μg/mL araliadiol treatment (141.18%) were reduced to 76.59% with GW9662 and to 72.98% with SB202190 (Figure 10B). These results indicate that araliadiol enhances VEGF secretion via the p38/PPAR-γ signaling pathway. Collectively, our findings demonstrate that the hair growth-promoting and anagen-inductive effects of araliadiol are regulated by the p38/PPAR-γ signaling pathway.

## 4 Discussion

In this study, we investigated the pharmacological effects of araliadiol as a novel treatment for hair loss. Our results demonstrated that araliadiol increased the levels of key proteins that induce hair growth in hair follicle cells. Notably, araliadiol enhanced the protein expression of VEGF, a crucial factor in hair growth, and upregulated the p38/PPAR-γ signaling pathway as its upstream mechanism. These findings suggest, for the first time, araliadiol, a polyacetylene compound, promotes hair growth and prevents hair loss, indicating its potential as a novel treatment for alopecia.

Our results demonstrated that araliadiol exerted proliferation-promoting effects on hair follicle cells within non-toxic ranges. Specifically, araliadiol treatment increased the protein expression of cyclin B1 and Ki67 in both cells. The promotion of hair follicle cell proliferation is known as a key biomarker for hair growth (Madaan et al., 2018; Kim et al., 2019). Because the proliferation and activation of DPCs and HFSCs are prerequisites for inducing the anagen phase of hair follicles, several previous studies have focused on identifying substances promoting the proliferation of hair follicle cells (Chi et al., 2010; Choi et al., 2013; Rastegar et al., 2015; Yoon et al., 2019).

We next confirmed whether araliadiol not only induced the proliferation of hair follicle cells but also increased different anagen signals promoting hair growth. We found that araliadiol dose-dependently increased the protein expression of VEGF, the most widely studied molecule that induces hair follicle growth (Yano et al., 2001). Specifically, in HHFSCs, araliadiol increased the expression of K15 and K19, markers specific to HFSCs, as well as CD34 and p63α, biomarkers known to be elevated in anagen hair follicles (Xing et al., 2024). Araliadiol also significantly increased the expression of VEGF and ANGPTL4, two proteins essential for hair follicle regeneration (Cazzola and Perez-Moreno, 2022; Kim and Sung, 2023). ANGPTL4, in particular, is known to increase its expression in bulge stem cells during the anagen phase, inducing the telogen to anagen transition in HFSCs (Gur-Cohen et al., 2019). In addition, araliadiol enhanced the paracrine factors known to promote hair follicle growth, such as FGF7, VEGF, IGF-1, and NOG, in HDPCs. These secretory factors, produced by DPCs, induce the proliferation and activation of HFSCs, promoting their differentiation into new hair shafts (Botchkarev et al., 2001; Yano et al., 2001; Hsu et al., 2011; Niu et al., 2023). Therefore, these results indicate that araliadiol exerts significant *in vitro* hair growth-promoting effects by upregulating anagen-inductive signals in both types of hair follicle cells.

PPAR is a member of the nuclear hormone receptor superfamily, with PPAR-α, δ, and γ distinctly expressed in human hair follicle cells (Billoni et al., 2000). Among the members of the PPAR family, PPAR-γ has been most extensively studied for its role in hair follicle growth and homeostasis maintenance. Deletion of the *PPAR-γ* gene has been reported to induce hair loss by reducing the expression of transcription regulators necessary for hair follicle development (Sardella et al., 2018). Our data demonstrated that the significantly increased anagen-inducing factor VEGF is upregulated by PPAR-γ (Chintalgattu et al., 2007; Forootan et al., 2016). Thus, we checked whether the hair growth-promoting effects induced by araliadiol were due to the activation of PPAR-γ signaling. Araliadiol increased the protein expression of PPAR-α and PPAR-γ among the PPAR family, with the most significant increase observed in PPAR-γ. As a transcription factor, the nuclear translocation of PPAR-γ, rather than its total protein level, is crucial. Therefore, we separated the nuclei and cytoplasm of hair follicle cells treated with araliadiol to confirm the protein expression of PPAR-γ. Our results revealed a significant increase in the nuclear expression of PPAR-γ in both HHFSCs and HDPCs treated with araliadiol. These findings suggest that, as in previous studies, araliadiol promotes the transcriptional activity by increasing the nuclear translocation of PPAR-γ (Kawai et al., 2010; Xu et al., 2010).

The activation of PPAR-γ as a transcription factor is influenced by the stability of the protein through different post-translational modifications (PTMs) (Han et al., 2013). Given the well-known short half-life of PPAR-γ, numerous efforts have been made to enhance its stability and thereby induce its transcriptional activity (Floyd et al., 2008; Ahmadian et al., 2013). PPAR-γ is primarily degraded by the ubiquitin-proteasome system, which regulates its protein homeostasis. Thus, inhibiting the proteasomal degradation of PPAR-γ can increase its protein half-life and enhance its transcriptional activity (Han et al., 2013; Noh et al., 2020). Our results indicated that araliadiol increased the total protein expression and nuclear translocation of PPAR-γ without affecting its mRNA expression. Therefore, we determined whether araliadiol increased the stability of PPAR-γ by inhibiting its proteasomal degradation. When treated with CHX, a protein synthesis inhibitor, the reduced protein expression of PPAR-γ was increased with the co-treatment of CHX and araliadiol. This confirmed that araliadiol increased the stability of PPAR-γ without affecting its mRNA expression. In addition, the expression of PPAR-γ increased in HHFSCs and HDPCs treated with the proteasomal inhibitor MG132. However, co-treatment with MG132 and araliadiol did not further increase the protein expression, suggesting that araliadiol inhibits the proteasomal degradation of PPAR-γ. Previous studies have demonstrated that increasing the protein stability of PPAR-γ can induce its transcriptional activation and upregulation of its downstream targets (Fei et al., 2012; Kumar et al., 2024). Therefore, the inhibition of PPAR-γ proteasomal degradation by araliadiol, along with an increase in total protein, supports the enhancement of PPAR-γ transcriptional activity.

Recent studies have revealed that the activation of PPAR-γ can promote hair growth and alleviate hair loss (Mirmirani and Karnik, 2009; Kim and Sung, 2023). Therefore, we assessed whether the increased anagen-inductive signals and PPAR-γ protein stability induced by araliadiol were related. Treatment with GW9662, a PPAR-γ antagonist, significantly decreased the PPAR-γ protein levels. In human hair follicle cells treated with both araliadiol and GW9662, the levels of K15, VEGF, and ANGPTL4 in HHFSCs and FGF7, VEGF, and NOG in HDPCs decreased to levels similar to those in the negative control. Thus, these findings demonstrate that araliadiol enhances anagen signaling by upregulating the stability of the PPAR-γ protein.

The protein stability of PPAR-γ can be regulated by several kinases such as ERK, p38, PKA, and JNK (Hedvat et al., 2004; Schild et al., 2006). Our results demonstrated a dose-dependent increase in the phosphorylation levels of p38, among the four kinases involved in stabilizing PPAR-γ. Previous studies have reported that ERK increases the phosphorylation of PPAR-γ at Ser112, promoting its proteasomal degradation. However, although p38 is part of the same MAPK family, it has been reported to inhibit PPAR-γ′s proteasomal degradation (Schild et al., 2006; Sangphech et al., 2020). Notably, studies by Schild have demonstrated that p38 does not significantly affect PPAR-γ mRNA but upregulates PPAR-γ protein expression and activity, which aligns with our results (Schild et al., 2006). Although this study did not demonstrate that p38 directly regulates the phosphorylation levels of PPAR-γ and blocks proteasomal degradation, numerous studies have reported that the activation of p38 can increase the phosphorylation levels of peroxisome proliferator-activated receptor gamma coactivator 1-alpha (PGC-1α), thereby enhancing the stability of PPAR-γ (Fei et al., 2012; Wang et al., 2016). This evidence suggests that the activation of p38 can directly or indirectly upregulate the activity and protein levels of PPAR-γ. Therefore, we used SB202190, a p38 inhibitor, to verify if the activation of p38 was responsible for the increased stability of PPAR-γ and hair growth-promoting signals. We found that araliadiol increased the phosphorylation level of MAPKAPK2 at T334, a downstream target of p38, whereas SB202190 alone significantly reduced this phosphorylation level. Moreover, in HHFSCs and HDPCs co-treated with both araliadiol and SB202190, the phosphorylation of MAPKAPK2 induced by araliadiol was reduced to levels comparable to the negative control. Similarly, the increased PPAR-γ and hair growth-inductive signals induced by araliadiol were inhibited in cells co-treated with SB202190, confirming that the *in vitro* hair growth-promoting effects of araliadiol depend on p38/PPAR-γ signaling.

From a chemical structural perspective, araliadiol is a polyacetylene compound belonging to the diene-diyne class, characterized by two carbon-carbon double bonds and two carbon-carbon triple bonds (Cheng et al., 2011). Polyacetylenes are widely found in biological systems and exhibit diverse biochemical activities influenced by factors such as chain length, degree of unsaturation, triple bond positioning, and the presence of functional groups (Christensen, 2010). Notably, the alkynyl group in polyacetylenes can act as a Michael acceptor in Michael addition reactions, showing strong affinity for biological proteins and facilitating various biological responses (Chen et al., 2015; Singh et al., 2020). For instance, polyacetylenes with conjugated triple bonds are known to interact with nucleophilic groups, such as thiols (Christensen, 2010). Additionally, oxylipins, another type of polyacetylenes, can form covalent bonds with cysteine residues in mitochondrial aldehyde dehydrogenase (ALDH), thereby reducing enzyme activity (Heydenreuter et al., 2015). Previous studies, including competitive ligand displacement assays and molecular docking analyses, have shown that polyacetylenes such as falcarinol, panaxydol, and falcarindiol can bind to human serum albumin (Wang et al., 2018). Moreover, falcarindiol has been identified as a ligand for PPAR-γ, forming hydrophobic interactions and hydrogen bonds with Cysteine 285 and Glutamate 295 residues (Atanasov et al., 2013). In this study, we demonstrated that araliadiol promotes hair growth at the cellular level by activating PPAR-γ signaling via p38 phosphorylation. However, further investigation is needed to identify the specific upstream protein target through which araliadiol regulates p38. Based on previous research, we hypothesize that the conjugated triple bonds in araliadiol may enable interactions with cysteine residues or hydrophobic interactions with p38 or its upstream regulatory proteins, thereby contributing to its biological activity.

Our study also revealed that araliadiol exhibits a biphasic effect, promoting cell proliferation at lower concentrations while showing cytotoxic effects at higher concentrations. The IC_50_ values were determined to be 13.750 μg/mL for HHFSCs and 21.831 μg/mL for HDPCs, aligning with previous findings for the polyacetylene compound falcarinol. Falcarinol was reported to stimulate cell proliferation at low concentrations (0.004–0.4 μM) but exhibited toxicity at concentrations above 4 μM (Hansen et al., 2003). This biphasic effect is consistent with the concept of hormesis, which posits that even potentially toxic compounds at high doses can have beneficial effects at low doses (Mattson, 2008). Although araliadiol showed cytotoxicity at higher concentrations, it is important to highlight that the risk of reaching toxic levels *in vivo* is likely minimal due to the low bioavailability of similar polyacetylenes. For instance, after the consumption of carrot juice containing 12.0 mg of falcarinol, blood concentrations reached only 0.0023 μg/mL and 0.0020 μg/mL after 2 and 5 h, respectively (Christensen, 2010). Additionally, the cytotoxicity of polyacetylenes at higher concentrations is well-documented to be closely linked to their chemical structure. Prior studies have indicated that the π-bond conjugation system and the geometry of double bonds are critical determinants of toxicity, with terminal hydroxyl groups and allylic alcohols, potentially contributing to their adverse effects (Ohta et al., 1999; Uwai et al., 2000). In conclusion, although araliadiol may induce cytotoxicity at higher concentrations, previous studies on similar polyacetylenes suggest minimal potential for *in vivo* toxicity. Further *in vivo* studies are necessary to establish pharmacologically effective concentrations that balance efficacy and safety. Additionally, the development of araliadiol derivatives with structural modifications to reduce toxicity could enhance its potential as a lead compound for future drug development, providing a pathway toward safer and more effective therapeutic options.

Overall, our study demonstrated that araliadiol promotes hair growth in an *in vitro* model and elucidated its molecular mechanism. Our findings align with previous *in vivo* studies showing that upregulation of the p38/PPAR-γ signaling pathway enhances hair growth in mice (Kim and Sung, 2023). Additionally, we observed that araliadiol increased VEGF levels through activation of the p38/PPAR-γ signaling pathway, producing effects comparable to minoxidil, a widely used treatment for both androgenetic alopecia and alopecia areata. These results suggest that, like minoxidil, araliadiol has the pharmacological potential to promote hair growth and could be considered for the treatment of various forms of alopecia. Future research should focus on validating the hair growth-promoting effects of araliadiol in preclinical studies using mouse models and in clinical trials.

## Data Availability

The original contributions presented in the study are included in the article/Supplementary Material, further inquiries can be directed to the corresponding author.
