# Supplementary figures and images for: In vitro hair growth-promoting effects of araliadiol via the p38/PPAR-γ signaling pathway in human hair follicle stem cells and dermal papilla cells
Source: Front Pharmacol. 2024 Dec 3;15:1482898. doi: 10.3389/fphar.2024.1482898 (PMC11649413; doi:10.3389/fphar.2024.1482898)

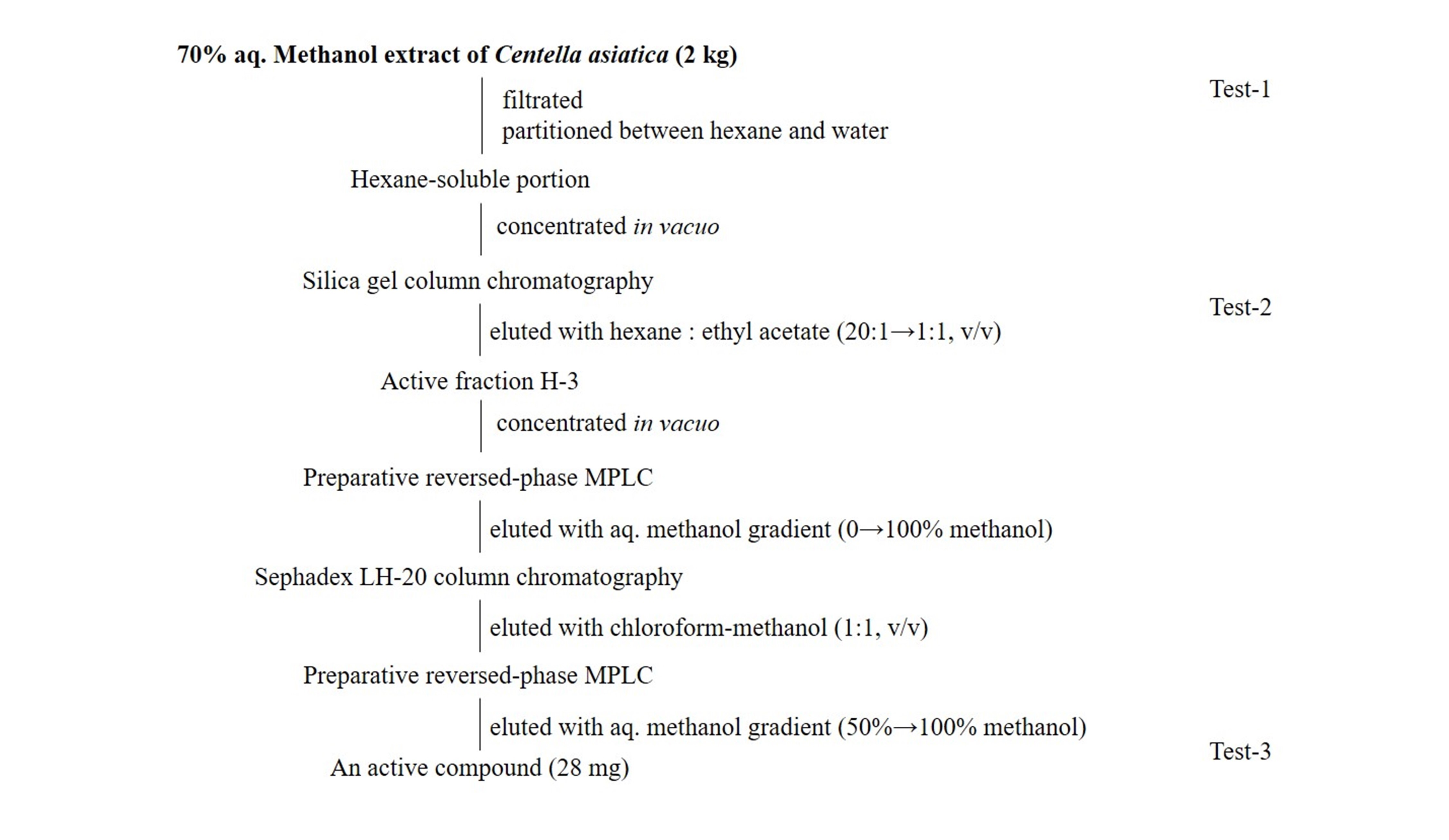

Supplement: Supplementary file 1 [file Image1.jpeg]

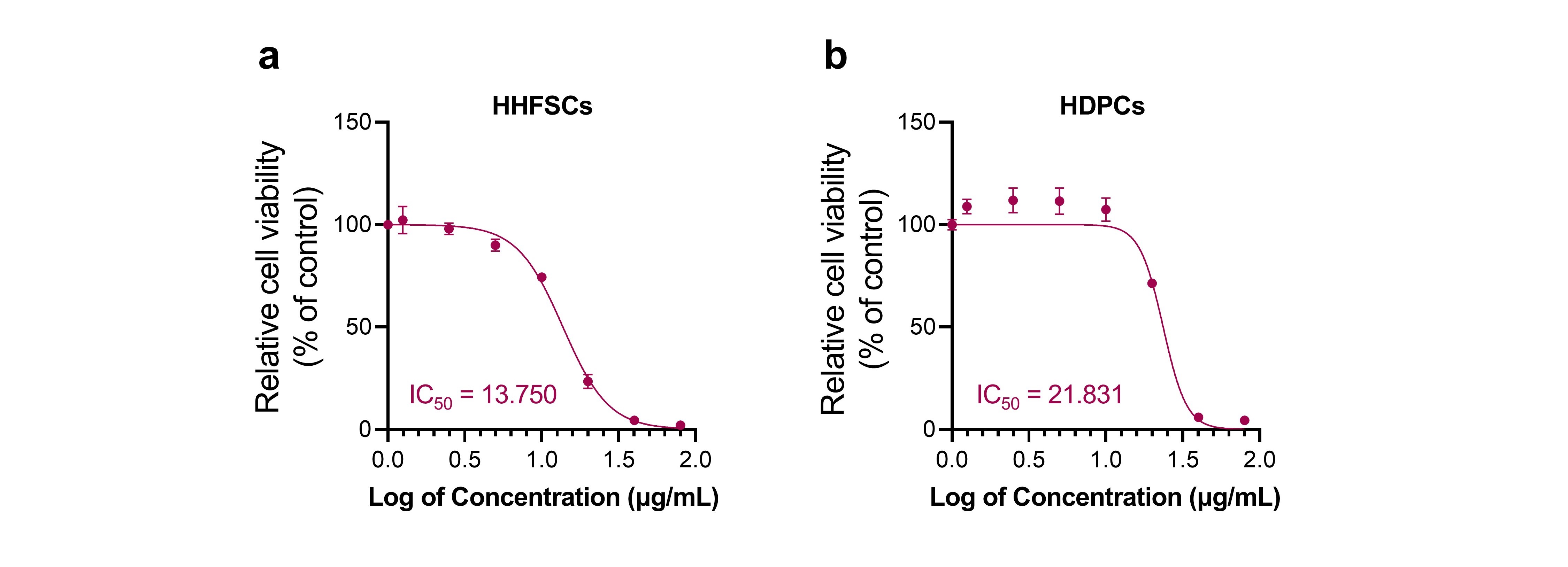

Supplement: Supplementary file 2 [file Image2.jpeg]
