# Supplementary material for: In vitro hair growth-promoting effects of araliadiol via the p38/PPAR-γ signaling pathway in human hair follicle stem cells and dermal papilla cells
Source: Front Pharmacol. 2024 Dec 3;15:1482898. doi: 10.3389/fphar.2024.1482898 (PMC11649413; doi:10.3389/fphar.2024.1482898)
Supplement: Supplementary file 3 [file DataSheet1.docx]

**Supplementary Material**


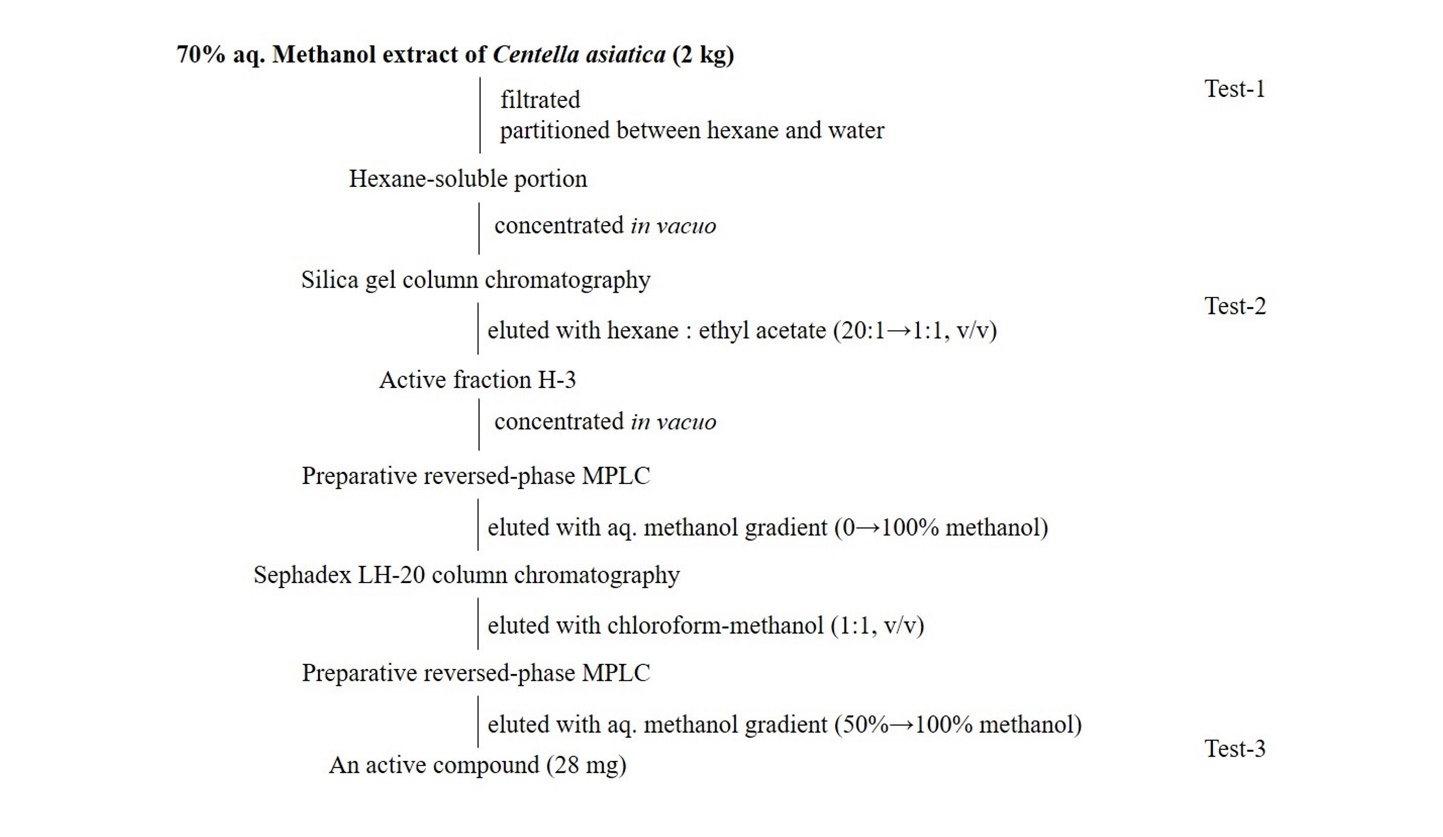


**Figure S1.** Isolation procedure of an active compound from *C. asiatica* extract.


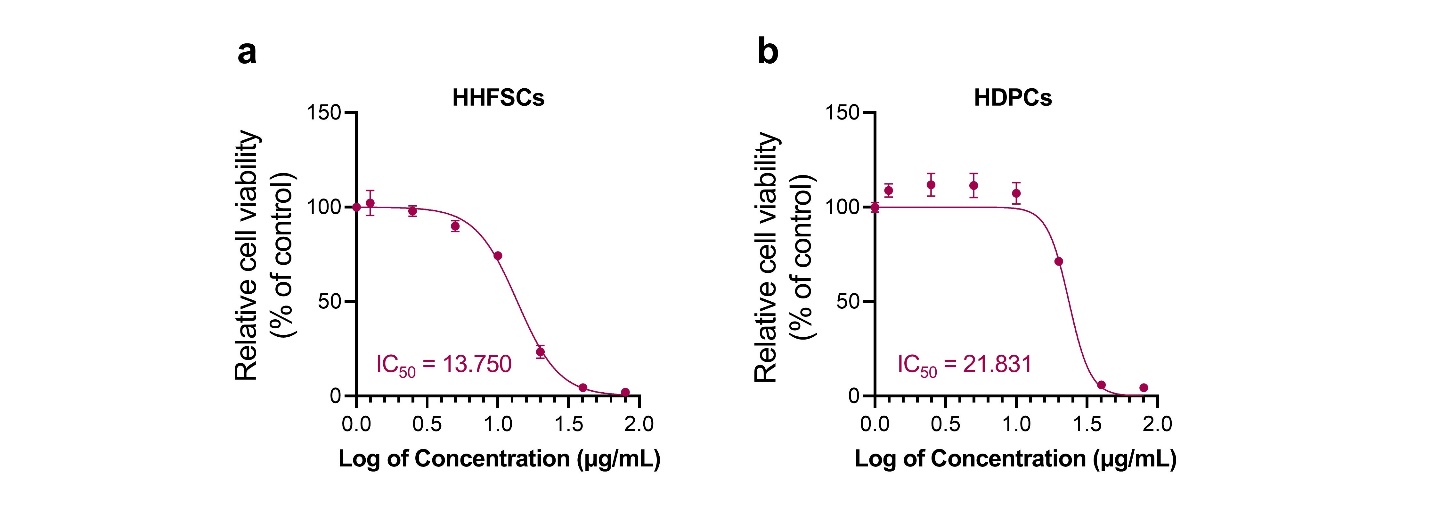


**Figure S2.** Dose-response curve and IC_50_ calculation of araliadiol on cell viability in human hair follicle cells.

**(a-b)** Human hair follicle cells were treated with varying concentrations of araliadiol (0-80 μg/mL) for 24 h. Cell viability in HHFSCs **(a)** and HDPCs **(b)** was assessed using a WST-1 assay. Results are presented as the mean ± SD of three independent experiments.
